# Lysine deserts and cullin-RING ligase receptors: Navigating untrodden paths in proteostasis

**DOI:** 10.1016/j.isci.2023.108344

**Published:** 2023-10-28

**Authors:** Natalia A. Szulc, Małgorzata Piechota, Lilla Biriczová, Pankaj Thapa, Wojciech Pokrzywa

**Affiliations:** 1 Laboratory of Protein Metabolism, International Institute of Molecular and Cell Biology in Warsaw, 4 Ks. Trojdena Str., 02-109 Warsaw, Poland

**Keywords:** Enzymology, Protein, Properties of biomolecules, Molecular interaction, Evolutionary biology

## Abstract

The ubiquitin-proteasome system (UPS) governs the degradation of proteins by ubiquitinating their lysine residues. Our study focuses on lysine deserts - regions in proteins conspicuously low in lysine residues – in averting ubiquitin-dependent proteolysis. We spotlight the prevalence of lysine deserts among bacteria leveraging the pupylation-dependent proteasomal degradation, and in the UPS of eukaryotes. To further scrutinize this phenomenon, we focused on human receptors VHL and SOCS1 to ascertain if lysine deserts could limit their ubiquitination within the cullin-RING ligase (CRL) complex. Our data indicate that the wild-type and lysine-free variants of VHL and SOCS1 maintain consistent turnover rates, unaltered by CRL-mediated ubiquitination, hinting at a protective mechanism facilitated by lysine deserts. Nonetheless, we noted their ubiquitination at non-lysine sites, alluding to alternative regulation by the UPS. Our research underscores the role of lysine deserts in limiting CRL-mediated ubiquitin tagging while promoting non-lysine ubiquitination, thereby advancing our understanding of proteostasis.

## Highlights

• Extensive bioinformatic screens to analyze lysine deserts' prevalence and conservation
• Proteomes of bacteria utilizing pupylation harbor lysine deserts
• Lysine deserts in VHL & SOCS1 limit ubiquitination by cullin-RING ligase complex
• Non-lysine ubiquitination may play a role in governing the lysine-deficient proteome

## Graphical abstract

**Figure:**
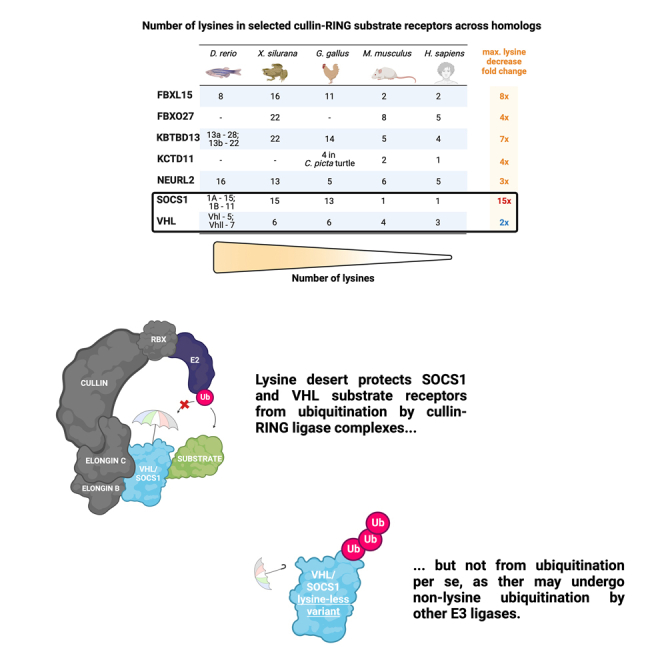

## Introduction

Protein homeostasis (proteostasis) stands central in ensuring cellular function and promoting organismal viability, a process prominently orchestrated via the ubiquitin-proteasome system (UPS).^1^^,^^2^ This intricate network operates through a hierarchical assembly of enzymes labeled as E1, E2, and E3, amongst which the E3 ligases take the pivotal role in ubiquitinating substrate proteins, thereby steering them toward varying fates.^3^^,^^4^^,^^5^ Mirroring the eukaryotic UPS, Actinobacteria employ a prokaryotic ubiquitin-like protein called Pup to modify target proteins through a process termed pupylation, showcasing an evolutionary conserved mechanism across biological kingdoms.^6^

Delving into the UPS further, the cullin-RING ligases (CRLs) emerge as the most substantial subgroup within the RING-type E3 family, supervising a noteworthy portion of ubiquitination events occurring in cellular environments.^7^ This process typically involves the modification of proteins with polyubiquitin chains, wherein different chain configurations can induce distinct outcomes, directing them toward proteasomal degradation or alternative pathways.^8^ Interestingly, aside from the conventional lysine residues, ubiquitination can occur at the N-terminus of a protein, as well as on serine, cysteine, and threonine residues, introducing a layer of complexity through non-lysine ubiquitination events, albeit with less thermodynamic stability.^9^^,^^10^

E3 ligases, known for their substrate specificity, are capable of ubiquitinating various positions within the "ubiquitination zone" of the substrate protein.^11^ Some E3s can initiate a process of auto-ubiquitination in the absence of substrate proteins, setting them on a pathway potentially leading to their own degradation.^12^ However, not all succumb to this fate; a range of strategies are deployed to bypass this automatic degradation route. They either engage with specific deubiquitinating enzymes (DUBs)^13^^,^^14^ or reduce lysine presence in crucial domains or sequences, reserving a scarce number for controlled ubiquitination. The latter is profoundly depicted in the structure of the yeast SUMO-targeted ubiquitin ligase Slx5, which retains a substantial lysine desert, constituting over 64% of its sequence, thereby effectively restricting its ubiquitin-dependent turnover.^15^ A similar regulatory mechanism is employed by the San1 yeast E3 ligase, where the absence of lysines in the disordered substrate-binding regions prevent immediate ubiquitination upon coupling with the E2-ubiquitin complex, thereby limiting self-destruction.^16^ This notion is further supported by the existence of lysine deserts in other protein quality control agents such as RAD23A, UBQLN1 or BAG6,^17^ and in many bacterial AB-type toxins that exhibit significant lysine depletion in their sequences possibly to avoid ubiquitination and subsequent degradation.^18^

Pursuing an understanding of the prevalence and evolutionary significance of lysine deserts, our study initiated extensive bioinformatic screenings across diverse prokaryotic and eukaryotic organisms. We explored the occurrence and characteristics of lysine deserts from bacteria leveraging the pupylation-dependent proteasomal degradation system to eukaryotes utilizing the UPS pathway, highlighting their functional and regulatory significance throughout evolutionary stages.

Focusing on human E3 ligase receptors: von Hippel-Lindau disease tumor suppressor (VHL) and suppressor of cytokine signaling 1 (SOCS1), we explored the potential of lysine deserts to curb their ubiquitination within their native CRLs. The wild type and lysine-deficient variants of VHL and SOCS1 constrain ubiquitination mediated by CRLs, maintaining steady turnover rates, and thereby hinting at a protective role for lysine deserts. Further, we unraveled their ubiquitination at non-lysine sites, suggesting alternate UPS-regulated pathways at play. Our study illuminates the potential of lysine deserts in protecting proteins within CRLs from undesirable ubiquitin tagging while also fostering non-lysine ubiquitination avenues, adding a new perspective to our understanding of proteostasis.

## Results

**Figure 1:**
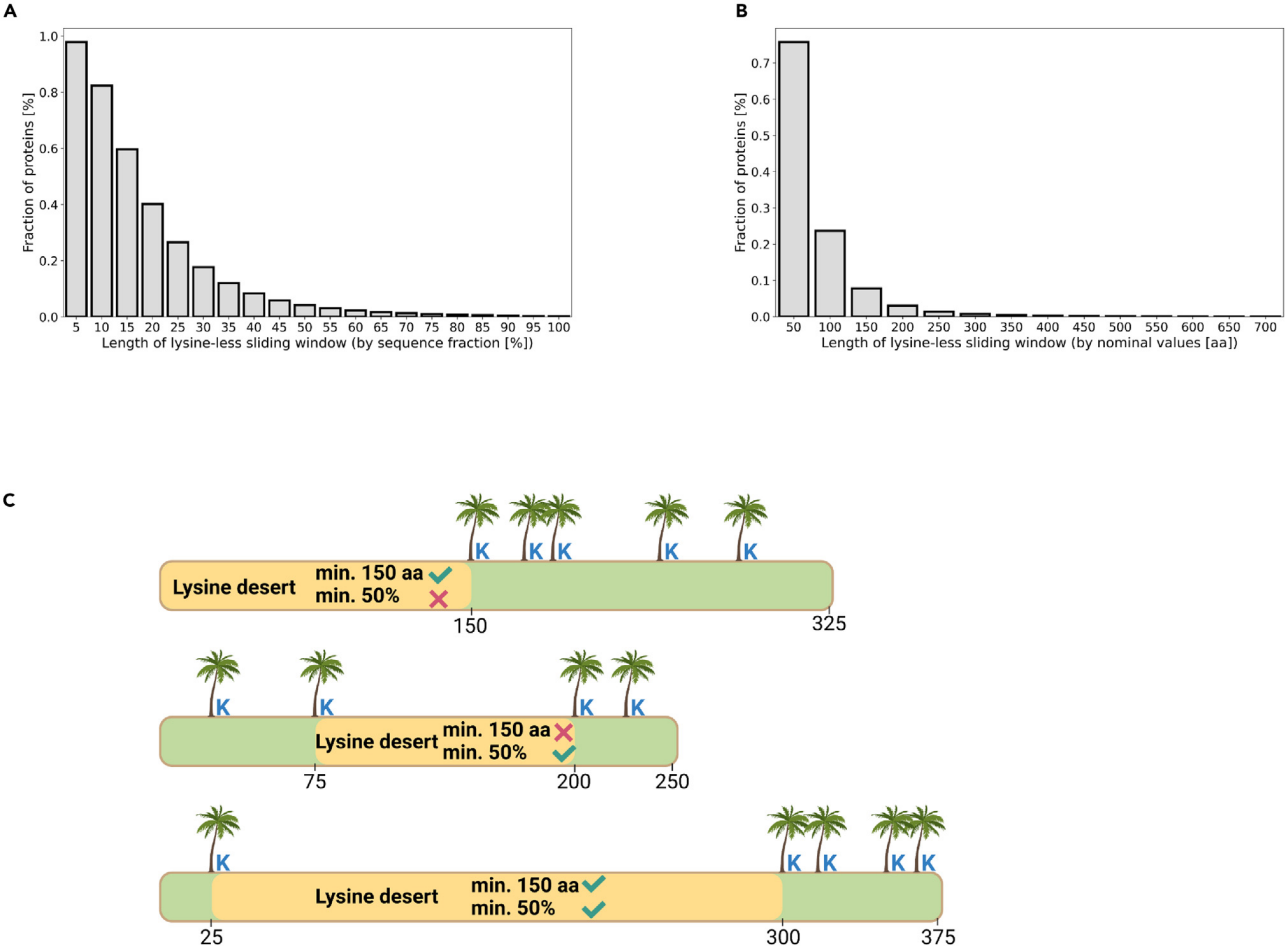
Definition of a lysine desert (A) Fraction of occurrence of lysine-less regions, probed by sliding windows of varying lengths defined as sequence fraction, among sequences (≥150 aa long) from all UniProt eukaryotic reference proteomes. (B) Fraction of occurrence of lysine-less regions, probed by sliding windows of varying lengths defined as the multiplicity of 50, among sequences (≥150 aa long) from all UniProt eukaryotic reference proteomes. (C) Two definitions of a lysine desert used in this work.

We first aimed to define the lysine desert region regarding the absolute amino acid (aa) length and sequence fraction. To find such thresholds, we searched for continuous lysine-less regions using sliding windows of varying lengths, defined as either sequence fraction (Figure 1A) or nominal value (Figure 1B), in proteins (≥150 aa) from all UniProt^19^ eukaryotic reference proteomes (1329 taxons; Table S1). Based on these results, we defined the lysine desert as a continuous lysine-less region either constituting min. 50% of a given sequence (hereafter lysine desert min. 50%) or min. 150 aa length (hereafter lysine desert min. 150 aa) (Figure 1C), as such defined lysine deserts occur in less than 10% of eukaryotic proteins.

### Lysine deserts are widespread among bacteria with pupylation pathway

**Figure 2:**
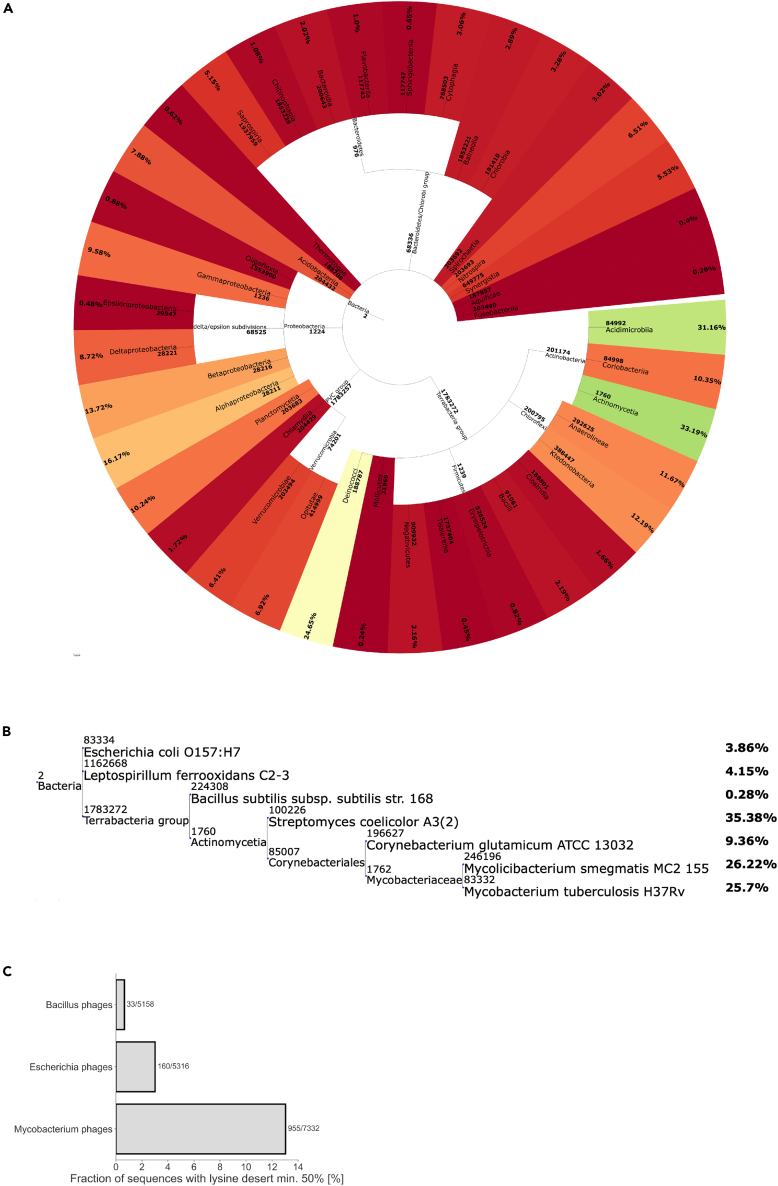
Lysine deserts in bacteria are most prevalent in *Actinobacteria* and their phages (A) Phylogenetic tree of different bacteria classes with a calculated average protein fraction with lysine desert min. 50% in the proteomes of their member taxons. Only classes with at least 10 member taxons were considered. The color gradient corresponds to the min-max normalization where red denotes classes with the lowest average fraction of desert min. 50% and green with the highest. (B) Phylogenetic tree of selected bacteria taxons from distinct classes with a calculated fraction of proteins with lysine desert min. 50% in their proteomes. (C) Bar plot presenting the fraction of lysine desert min. 50% in pan proteome of selected phages’ groups. The number of sequences with lysine desert and the total number of analyzed sequences are indicated to the right of each bar.

Next, we aimed to ascertain if the lysine deserts may have already emerged in bacteria that employ a pupylation and proteasome-dependent degradation pathway. We analyzed all available bacteria reference proteomes (for 8881 taxons; Table S1) from the UniProt database to check the fraction of proteins (≥150 aa) possessing a lysine desert min. 50%/150 aa in each proteome separately and averaged the results across different taxonomic classes. Interestingly, *Actinobacteria* possess the most proteins with lysine desert min. 50% in their proteomes (Figure 2A), and the same tendency is preserved for lysine desert min. 150 aa (Figure S1A). We also compared proteomes of the most studied bacteria taxons belonging to different taxonomic classes - *M. tuberculosis* H37Rv (virulent), *M. smegmatis*, *C. glutamicum*, *S. ceolicolor*, *L. ferrooxidans*, *B. subtilis*, and *E. coli*. From each aforementioned proteome, we again selected sequences ≥150 aa, and with no more than two predicted transmembrane helices (TMH) using the TMHMM-2.0 software^20^^,^^21^; this condition was applied to exclude proteins with multiple transmembrane regions as they would introduce bias due to their reduced frequencies of polar residues.^22^ Applying these criteria resulted in analyzing 58–71% of sequences (see STAR Methods). Again, the most lysine desert-rich proteomes belonged to the *Actinobacteria* phylum that utilizes the pupylation route for protein breakdown, regardless of the applied lysine desert definition (Figures 2B and S1B). It is noteworthy that the number of sequences with lysine deserts min. 50% was approx. 3- to 4-fold higher in taxons that encode and use the proteasome for the regulated degradation of pupylated proteins^6^ (25.7% for *M. tuberculosis*, 26.2% for *M. smegmatis* and 35.4% for *S. ceolicolor*) than those that use Pup-modifications but lack proteasomal subunit genes^6^ (9.4% for *C. glutamicum*).

To further investigate the possible linkage of pupylation and lysine desert occurrence, we retrieved information on identified pupylated proteins (pupylomes) of *M. tuberculosis*, *M. smegmatis,* and *C. glutamicum* from the PupDB database,^23^ which gathers data from four large-scale proteomics studies. Interestingly, only about 1% of these bacterial species' whole proteomes (unfiltered in any way) undergo pupylation (Table S2). As previously, we selected sequences ≥150 aa with no more than two predicted TMH among pupylated and non-pupylated proteins of the taxons mentioned above (as non-pupylated we considered all proteins in a proteome except those reported in the PupDB) and searched for lysine deserts among them. We noted that the fraction of sequences with lysine desert min. 50% among pupylated proteins was 1.52% for *M. smegmatis*, 2.56% for *C. glutamicum*, and 8.16% for *M. tuberculosis* (Table S3). The presence of proteins with lysine desert min. 150 aa was also much more prevalent in *M. tuberculosis* (Table S4). Notably, this trend was not related to the average length of filtered sequences in both analyses. In line with the previous results, the fraction of sequences with lysine desert among non-pupylated proteins was approx. 3- (lysine desert min. 50%) to 4- (lysine desert min. 150 aa) fold higher in bacteria employing proteasome to protein turnover (Tables S5 and S6). This observation suggests an as-yet-unidentified mechanism of Pup avoidance on lysines and, thus, proteasomal degradation, similar to yeast examples.^15^^,^^16^

### *Mycobacterium* phages are equipped with lysine desert proteins

Phages are known to be highly specific toward their hosts, co-evolving with them to adopt successful hijacking strategies. Therefore, we decided to inspect whether the difference in lysine desert quantity between *Actinobacteria* and other bacteria phyla also finds reflection in phages specific to different genuses of bacteria. We selected 133 to 527 proteomes of phages specific to *Bacillus*, *Escherichia*, and *Mycobacterium* genuses and clustered them into separate pan proteomes containing 5158 to 7332 sequences ≥150 aa (see STAR Methods). Interestingly, sequences of *Mycobacterium* phages contain approx. 4- (lysine desert min. 50%) to 5- (lysine desert min. 150 aa) fold more proteins with lysine desert compared to *Escherichia* phages and from approx. 12- (lysine desert min. 150 aa) to 22- (lysine desert min. 50%) fold more proteins with lysine desert compared to *Bacillus* phages (Figures 2C and S1C). This observation suggests an adaptive strategy by phages to minimize the pupylation of viral proteins, potentially avoiding their removal by the host cell.

### Lysine deserts appear with increasing organismal complexity in eukaryotes

**Figure 3:**
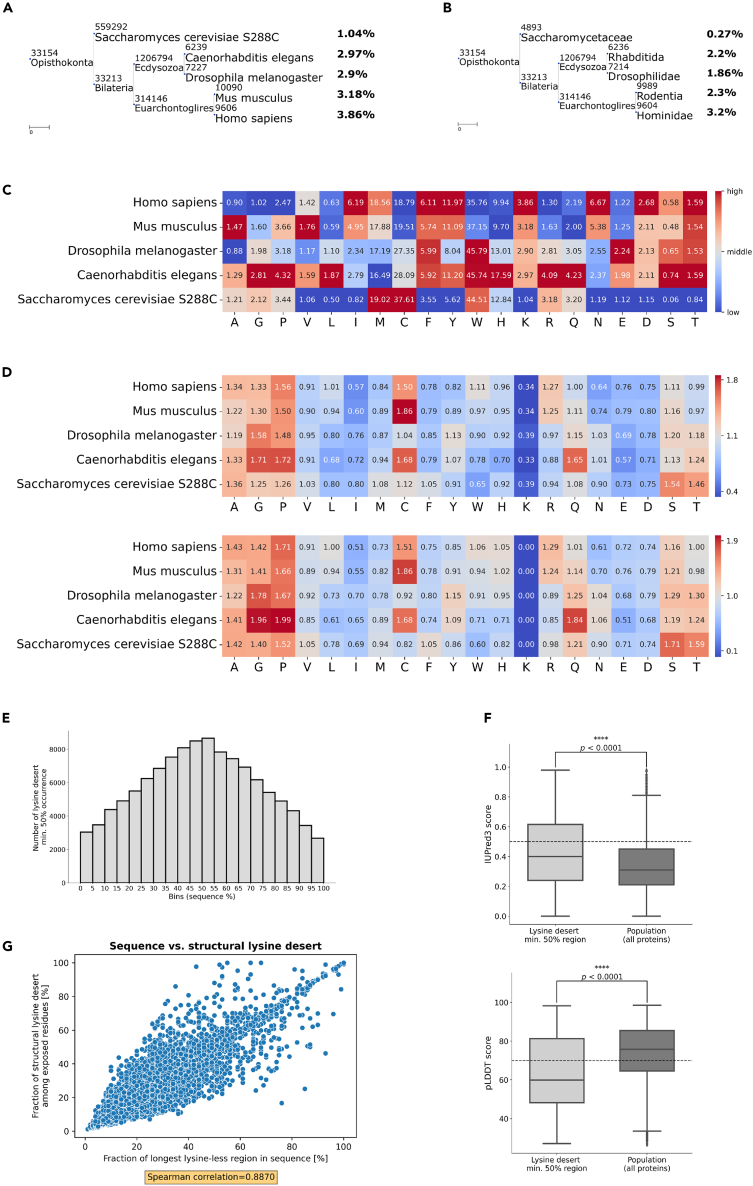
Lysine deserts in eukaryotes ascend with growing organismal complexity (A) Phylogenetic tree of selected eukaryotic model organisms with a calculated protein fraction with lysine desert min. 50% in their proteomes. (B) Phylogenetic tree of selected eukaryotic families/order, which include model organisms used in the previous analysis, with calculated fractions of conserved lysine desert min. 50% among their OGs. (C) Heatmap of fractions of proteins with desert region min. 50% of each of 20 aa among proteomes of selected eukaryotic model organisms. (D) Heatmaps of relative fractions of each amino acid in sequences of selected eukaryotic model organisms with a lysine desert min. 50% normalized to the entire appropriate proteome; value of 1.00 indicates no change. Up - considering whole sequences; down - considering only the lysine desert regions. (E) Histogram of distribution of sequence location of lysine desert min. 50% regions in the human proteome. (F) Boxplots of predicted disorder score of residues constituting lysine desert regions min. 50% only vs. residues of all proteins in the human proteome. Up - sequence-based disorder predictions obtained using the IUPred3 software; higher values indicate a higher disorder probability. Down - structure-based disorder predictions based on the pLDDT values obtained from the AlphaFold2 models of the human proteome; lower values indicate a higher probability of disorder. Disorder cut-offs proposed by the authors of the methods are marked with dashed lines. The stars denote the significance levels per two-tailed p value obtained from the Mann-Whitney U rank test. (G) Scatterplot showing co-occurrence of sequence and structural lysine deserts in human proteome based on the AlphaFold2 models. The Spearman rank-order correlation coefficient is denoted below the plot.

We carried out a screen for proteins with lysine desert regions among the proteomes of five model eukaryotic organisms: S*. cerevisiae*, *C. elegans*, *D. melanogaster*, *M. musculus*, and *H. sapiens*. Again, we excluded very short sequences (<150 aa) with more than two TMH predicted by the TMHMM-2.0 software; hereafter, we refer to those as filtered proteomes. This filtering procedure resulted in the analysis of 66–77% of the sequences (see STAR Methods). We observed an ascending trend of lysine desert proteome coverage - fractions of lysine desert proteins constituted from 1.04%/2.38% of *S. cerevisiae* proteome to 3.86%/10.5% of *H. sapiens* proteome (lysine desert min. 50%/min. 150 aa, respectively) (Figures 3A and S2A).

Next, we assessed whether the lysine desert regions are conserved among closely related orthologs. We conducted a similar analysis of lysine desert coverage within all available Orthologous Groups (OGs; each OG contains sequences of analogous proteins) from the eggNOG5^24^ database of different taxa, which include model organisms used in the above described analysis. As previously, we excluded OGs of short, transmembrane, or unrepresentative proteins (see STAR Methods for details). Again, we noticed that with the increase of organismal complexity, fractions of OGs with conserved lysine desert proteins were ascending, constituting 0.27%/1.04% of OGs in *Saccharomycetaceae* to 3.2%/9.72% in *Hominidae* (lysine desert min. 50%/min. 150 aa, respectively) (Figures 3B and S2B). These results indicate that lysine desert fractions expand with increasing organismal complexity, and these regions are conserved in homologous proteins, indicating their potential functional involvement.

### Evolutionary depletion of lysines is unique among positively charged amino acids

We evaluated the uniqueness of the observed tendency of lysine desert ascendance concerning the amino acid whose absence establishes the desert region. For this purpose, we searched for regions devoid of each of the 20 amino acids in the filtered proteomes of the same organisms. We detected a similar gradient trend in isoleucine, asparagine, and aspartic acid for the desert min. 50% (Figure 3C) and additionally in tyrosine and threonine for the desert min. 150 aa (Figure S2C). Interestingly, there was no similar evolutionary ascendance of arginine or histidine desert regions, although these residues, similarly to lysine, yield a positive charge; in fact, we detected a reversed trend for arginine.

We then aimed to determine whether lysine desert proteins are enriched or depleted in particular amino acids. We calculated the relative frequencies (each amino acid was normalized by its frequency in the population of a given filtered proteome) of 20 amino acids among the lysine desert proteins and their lysine desert region only in the same filtered proteomes. Arginine was moderately enriched in *M. musculus* and *H. sapiens* among proteins with lysine desert min. 50% but not min. 150 aa. This may indicate that lysine-less regions could compensate for the lack of lysine with another positively charged amino acid, arginine. We also observed that proteins with lysine desert min. 150 aa and lysine desert min. 50% are enriched (19%–116%) in alanine, glycine (except for *S. cerevisiae*), and proline in all analyzed eukaryotic model organisms (Figures 3D and S2D), which may point to the occurrence of low complexity regions within them.^25^

To obtain a picture of the preferred location of the lysine desert regions, we analyzed their distribution among all human protein sequences (again, when referring to the human proteome, we mean its filtered version as described previously; data on lysine desert occurrence of a given type in each human protein are available in Table S7). Interestingly, regions of lysine desert min. 50% tend to occupy internal parts of the protein, avoiding the N-/C-terminus (Figure 3E). This tendency, albeit not as pronounced, is also evident for lysine desert defined as min. 150 aa (Figure S2E).

### Lysine deserts frequently appear on protein surfaces but may not align with the amino acids of lysine-deficient sequence regions

As lysine desert proteins possess the characteristic of low-complexity regions and lysine deserts of yeast San1 and Slx5 E3s are chiefly disordered, we endeavored to determine the structural features of lysine desert regions across the human proteome. For each protein, we predicted its disorder score based on its sequence using the IUPred3 software^26^ and obtained the pLDDT (predicted Local Distance Difference Test) values, which estimate the modeling accuracy of each residue,^27^ from the corresponding AlphaFold2 model^28^^,^^29^ (see STAR Methods). Both techniques have been demonstrated to be the gold standard for forecasting disordered regions.^26^^,^^30^ Using a sequence- and a structure-based method, our approach allowed us to get unbiased and consistent results - lysine desert regions, either min. 150 aa or min. 50%, are more disordered than the entire human proteome (Figures 3F and S2F).

Since protein structure constitutes the interface of intermolecular interactions and therefore plays an essential role in protein functioning, we aimed to evaluate whether lysine desert regions detected in sequences are also maintained in structures. To this end, we screened AlphaFold2 models of the human proteome^29^ to search for the structural lysine deserts. Of note, we did not use any cut-off values regarding its length as in the case of sequence lysine desert (Figure 1C); rather, we defined it as the most extended, uninterrupted lysine-less region among solvent-exposed residues remaining in contact (see STAR Methods for the details on our algorithm). Our analysis showed a strong correlation between the length of lysine-less regions in sequence and the structure of the human proteome (its filtered version; we also excluded from the analysis proteins with more than 5% of residues without calculated solvent accessibility values) (Figure 3G). Interestingly, the number of common residues between sequence/structural lysine desert often varies, with most pronounced cases where a protein with a lysine-less region spanning over 40% of its sequence shows no overlap with the structural lysine desert (Figure S2G). This indicates how important it is not to overlook the structures, as the information encoded in the sole sequence of a protein may provide a misinterpretation of data. Information on the length and residues building the three most extended structural lysine deserts, tabulated with information about the longest lysine-less regions present in the sequence, can be found in Table S8.

### Most conserved lysine desert proteins operate within the ubiquitin-proteasome system, often clustering their remaining lysines

**Figure 4:**
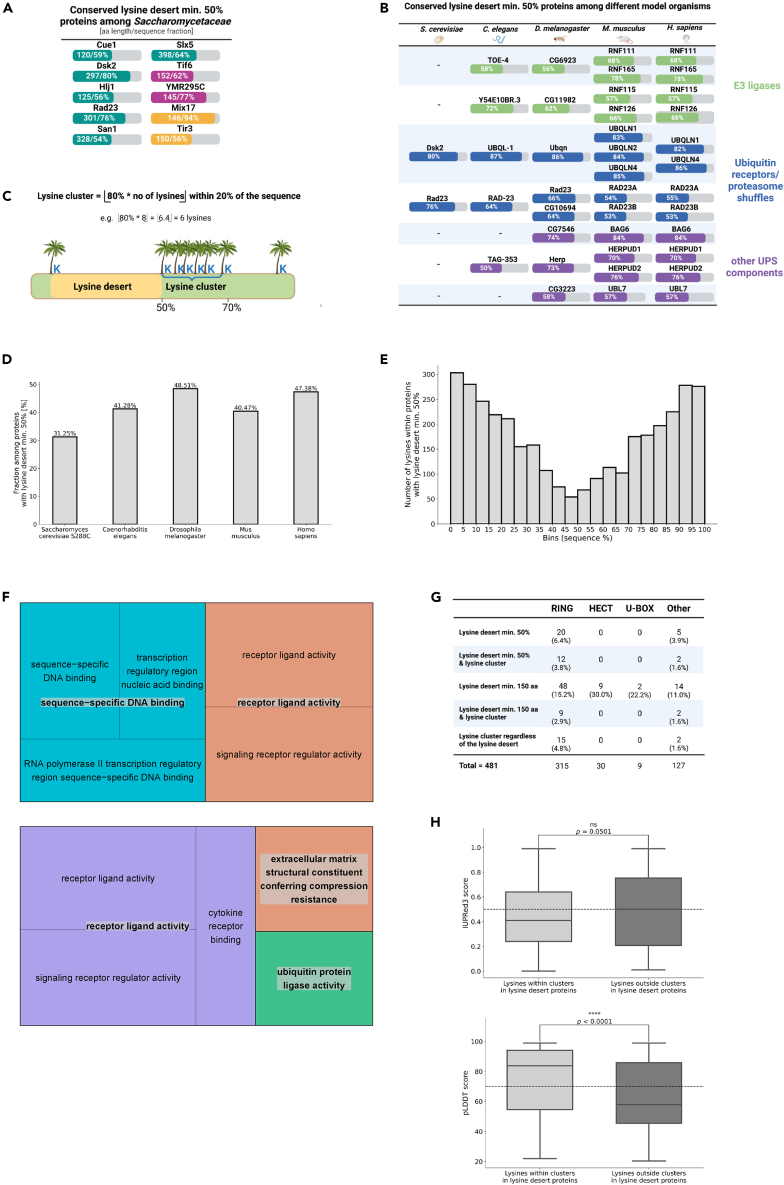
Lysine deserts are conserved among UPS proteins and often co-occur with a lysine cluster (A) Conserved lysine desert min. 50%-containing proteins among the *Saccharomycetaceae* family. Gene names are provided for *S. cerevisiae.* Nominal values of the lysine desert’s length and sequence fraction in each protein are denoted on the bars. Teal - proteins associated with protein turnover; magenta - ribosomal proteins; yellow - other proteins. (B) Proteins with the most evolutionarily conserved lysine deserts. The selection criteria were as follows: lysine desert min. 50% conserved in OGs from *Drosophilidae*, *Rodentia,* and *Hominidae* (in *Saccharomycetaceae* and *Rhabditida*, OGs were either absent or, if present, conserved lysine desert min. 50% also needed to occur). Each row represents one vector set of analogous OGs. For clarity, fractions of lysine desert regions in proteins from the model organism representative of the aforementioned families/order are shown. (C) Definition of a lysine cluster. (D) Bar plot of the fraction of lysine clusters’ occurrence among proteins with lysine desert min. 50% from selected model organisms. Exact values are denoted above each bar. (E) Histogram of distribution of sequence location of lysines within proteins with lysine desert min. 50% from the human proteome. (F) GO molecular function terms found to be associated with proteins with lysine desert min. 50% (up) and with proteins with lysine desert min. 50% co-occurring with a lysine cluster (down). (G) Summary of co-occurrence of lysine deserts min. 150 aa and min. 50% and lysine clusters among different families of human E3s. (H) Boxplots of predicted disorder score of lysine residues belonging to a lysine cluster among proteins with lysine desert min. 150 aa and/or min. 50% vs. lysine residues, which do not form a lysine cluster among proteins with lysine desert min. 150 aa and/or min. 50% in the human proteome. Up - sequence-based disorder predictions obtained using the IUPred3 software; higher values indicate a higher disorder probability. Down - structure-based disorder predictions based on the pLDDT values obtained from the AlphaFold2 models of the human proteome; lower values indicate a higher probability of disorder. Disorder cut-offs proposed by the method’s authors are marked with dashed lines. The stars denote the significance levels per two-tailed p value obtained from the Mann-Whitney U rank test.

Functional lysine deserts were originally identified in yeast E3 ligases.^15^^,^^16^ Therefore, we decided to establish if lysine-free sequences are exclusive to the UPS pathway. To this end, we analyzed the function of lysine desert proteins and their evolutionary conservation among organisms with comparable and vastly different levels of complexity. First, we checked which proteins of *S. cerevisiae*, the simplest eukaryotes studied here, possess conserved lysine desert min. 50% among their orthologs in the *Saccharomycetaceae* family. We used the same set of OGs from *Saccharomycetaceae* and the methodology of defining a conserved lysine desert as previously described (see STAR Methods). As the criteria for recognizing OGs as conserved lysine desert-containing were very stringent, we found 10 such cases for the following proteins: Cue1, Mix17, Rad23, San1, Slx5, Tif6, Tir3, and YMR295C (gene names provided for *S. cerevisiae*) (see Table S9; summaries of OGs with conserved lysine desert min. 150 or 50% in *Saccharomycetaceae*, *Rhabditida*, *Drosophilidae*, *Rodentia*, and *Hominidae* are available at https://github.com/n-szulc/lysine_deserts^31^). Notably, six of the listed - Cue1, Dsk2, Hlj1, Rad23, San1, and Slx5, regulate protein turnover (Figure 4A).

Next, we aimed to compare the evolutionary conservation of lysine deserts min. 50% between analogous OGs of different eukaryotic families/orders (e.g., whether protein X with a conserved lysine desert in a given OG of *Saccharomycetaceae* also has a conserved lysine desert in an analogous OG of *Hominidae;* we term such analogous OGs as vectors). For this analysis, we examined the occurrence of conserved lysine deserts in the vectors of respective OGs (see STAR Methods for details; complete results are available at https://github.com/n-szulc/lysine_deserts)*.* We applied stringent criteria - wechecked for the presence of lysine desert min. 50% that would be conserved in any of 4282 OG vectors from *Drosophilidae*, *Rodentia,* and *Hominidae* (for *Saccharomycetaceae* and *Rhabditida*, OGs either could be absent or, if present, conserved lysine desert min. 50% also needed to occur). We again found proteins responsible for protein degradation (7 out of 9 vectors; see Table S10) - E3s, ubiquitin-adaptor proteins, and other proteostasis components (Figure 4B).

Interestingly, during our analyses we noticed that lysines in some lysine desert proteins (e.g., RNF126, RNF165, ubiquilins, HERPUD1, HERPUD2) occur within a relatively limited part of the sequence. We reasoned that such clustering of lysines, while maintaining a significant lysine desert region, could play a regulatory role in controlling their turnover. We estimated the frequency of lysine clusters within the lysine desert proteins to examine how this tendency relates to our global studies of selected model organisms’ proteomes (again, their filtered versions as previously described). We established a criterion to define a lysine cluster as ⌊80% ∗ total number of lysines in sequence⌋ confined within 20% of the sequence (applicable only to proteins with at least two lysine residues) (Figure 4C). This allowed us to identify proteins potentially utilizing bidirectional lysine signaling, characterized by extended lysine-free stretches coupled with lysine-rich regions concentrated in a specific part of the sequence, acknowledging that not every protein with a substantial lysine desert coincides with a lysine cluster. Over 47% of human lysine desert min. 50% proteins possess a lysine cluster, and other model organisms also show high (31–49%) such co-occurrence (Figure 4D). However, this trend is much less pronounced for lysine desert min. 150 aa, where lysine cluster occurs in 10–16% of such proteins from the analyzed model organisms (Figure S3A). In reference to the globally filtered proteomes, lysine clusters are found in 0.8–3.3% of proteins (Figure S3B), suggesting their rare occurrence but a higher prevalence in proteins featuring a sizable lysine desert within their sequence. We also analyzed lysines’ distribution among sequences of human proteins possessing lysine desert regions, finding that while lysine deserts favor the internal parts of the protein (Figures 3E and S2E), the remaining lysines display a bimodal distribution with peaks at N-/C-terminus (Figures 4E and S3C). Even among proteins with lysine desert min. 150 aa, which have a much flatter distribution of their lysine-less regions (Figure S2E), there is a visible tendency of lysines to localize at the C-terminus (Figure S3C).

To evaluate molecular functions associated with lysine deserts and lysine clusters, we performed Gene Ontology (GO)-based overrepresentation analysis of genes derived from sets of human proteins with lysine desert min. 50%. We observed that molecular functions over-represented among proteins possessing lysine desert min. 50% are broadly defined as DNA-binding and receptor-ligand signaling (Figure 4F, upper panel), indicating the diverse roles of such proteins. Likewise, for lysine desert defined as min. 150 aa, the molecular functions are also broadly annotated (Figure S3D). Noteworthy, for proteins where a lysine desert min. 50% co-occurs with a lysine cluster, ubiquitin ligase activity is over-represented (Figure 4F, lower panel), which may point to the functionality of this lysine division in the context of a vast lysine desert, especially within the ubiquitin-signaling components. The abovementioned results prompted us to look closely at the occurrence of lysine deserts and lysine clusters among E3 ligases. Given the discrepancies in the existing literature concerning the number of E3 ligases in the human proteome, we manually curated a list of 481 human proteins associated with ubiquitin ligase activity, inclusive of their family annotation (Table S11). We detected that 25 of them possess a lysine desert min. 50%, and within them, 14 also have a lysine cluster (Figure 4G). Interestingly, when we analyzed E3s by their types, it was remarkable that the lysine desert and lysine cluster are typical features of the RING E3 ligases - 12 out of 14 E3s with both lysine desert min. 50% and a lysine cluster were of this type; a similar trend also occurs for lysine desert min. 150 aa (Figure 4G). In addition, lysine-deficient regions are primarily found in the disordered regions of the human RING E3s (Figures S3E and S3F).

Similarly as for lysine deserts, we wanted to check whether lysine clusters co-occurring with lysine deserts tend to locate in disordered regions of proteins. Using the same approach as previously, we noted that lysines of lysine clusters are more structured than other lysines among human proteins with lysine desert (Figure 4H). Several human E3s, such as aforementioned highly conserved RNF126 and RNF165, but also other conserved among mammals, e.g., RNF6 (lysine desert of 527 aa, constituting 77% of the sequence), RNF12/RLIM (lysine desert of 477 aa, constituting 76% of the sequence), or RNF44 (lysine desert of 350 aa, constituting 81% of the sequence) aggregate their lysine residues within the RING domain, which interacts with E2s. Presumably, owing to the lysine clusters in the functional domains, E3 can undergo precise auto-ubiquitination without modification in the vast lysine desert region, which i.e., could affect substrate binding.

### Cullin-RING ligase substrate receptors are deprived of lysines in the course of evolution

**Figure 5:**
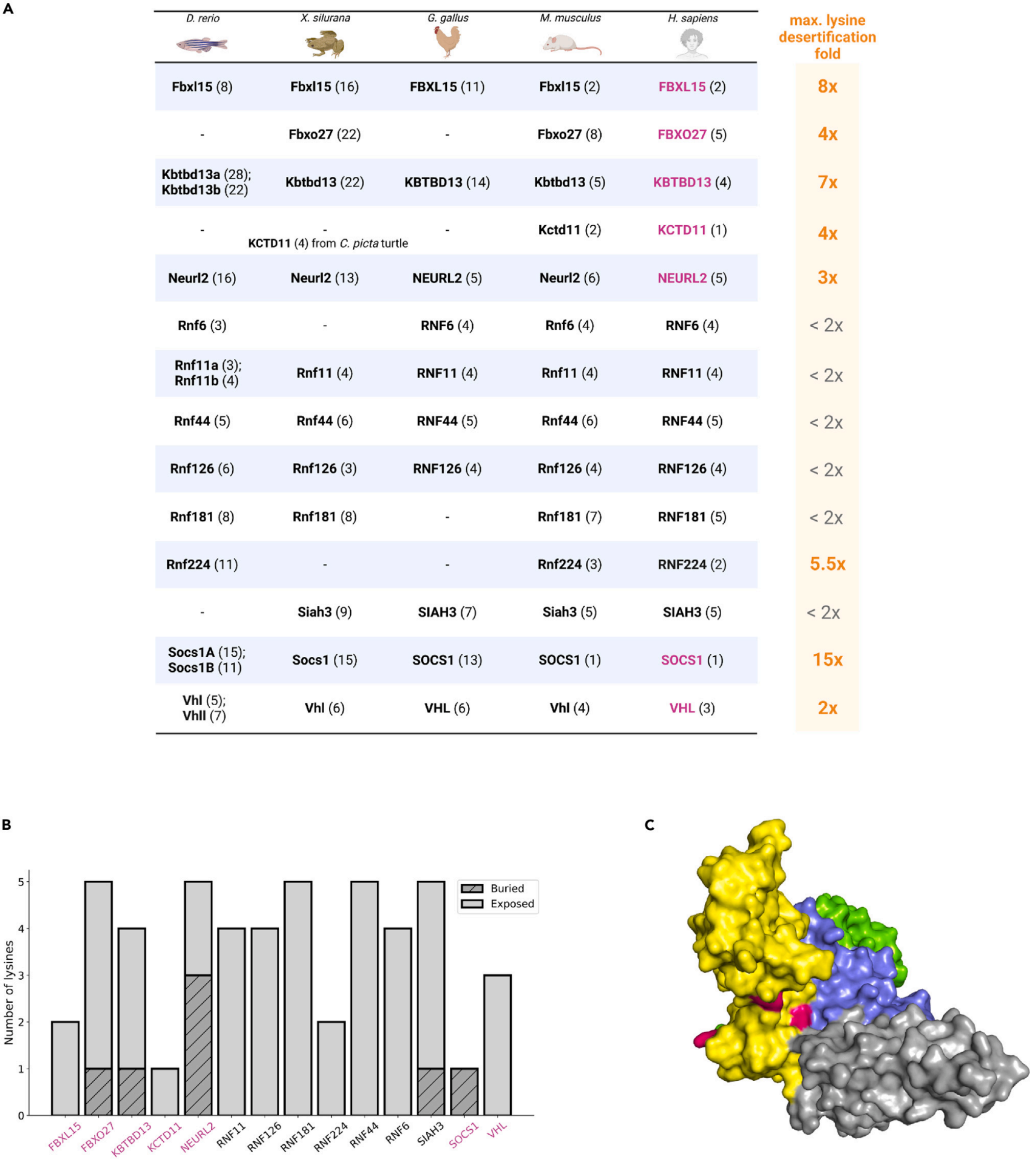
Human CRL substrate receptors are evolutionarily deprived of lysines and restrict solvent accessibility for the remaining ones (A) Human E3 ligases possessing max. five lysines and their orthologs from *D. rerio*, *X. silurana, G. gallus,* and *M. musculus*. A dash marks unknown orthologs; as a result of genome duplication in *D. rerio* its orthologs are separated with a semicolon. Due to unknown orthologs from *D. rerio*, *X. silurana,* and *G. gallus* of KCTD11, the number of lysines in its ortholog from *C. picta* turtle was reported. The total number of lysines for each protein is denoted in brackets. Human CRL substrate receptors are marked in magenta; maximum evolutionary “lysine desertification” is indicated to the right. (B) Bar plot of the number of exposed and buried lysines in human E3 ligases possessing max. five lysines. Relative solvent accessibility (RSA), based on which residue is classified as exposed or buried, was calculated using the DSSP program and the Sander method (see STAR Methods for details). Residues with RSA >0.2 are considered as solvent-exposed. Human CRL substrate receptors are marked in magenta. (C) VHL in complex with cullin 2 (PDB ID: 4WQO). Color codes explanation: yellow - VHL, green - elongin B, blue - elongin C, gray - cullin 2, magenta - lysine residues (three in total) of VHL. Visualized in the PyMOL software (Schrödinger) (v. 2.5.0).

The restricted availability of orthologous sequences from evolutionarily distant organisms (such as invertebrates and mammals) poses a challenge for comparative analyses. Moreover, searching only for long lysine-less regions may prevent the detection of proteins that lost lysines during evolution but do not have lysine-free stretches long enough to surpass the predetermined threshold (e.g., the threshold for considering protein as containing a lysine desert min. 50%). For these reasons, we searched for E3 ligases in the filtered human proteome (Table S7) possessing max five lysines, excluding those described in the UniProt database as membrane-bound (even single-pass). We tabulated the obtained lysine-poor E3s with their distant vertebrate orthologs from *D. rerio*, *X. tropicalis*, and *G. gallus,* as well as from more closely related *M. musculus,* based on the information from the eggNOG5 and Xenbase^32^ databases (Table S12). Out of 14 such human E3 ligases, eight showed significant “lysine desertification” (from 2- up to a 15-fold decrease of lysines) in the course of evolution (Figure 5A). The majority (seven out of eight) of such E3s act as substrate receptors subunits of CRLs. Within CRL complexes, while binding to client proteins and positioning them for ubiquitination by the E2 enzyme, these receptors are at risk of unintended ubiquitination. The presence of a lysine desert may thus serve a functional role in mitigating this modification.

Since lysine is one of the most solvent-accessible amino acids,^33^ we next aimed to determine if the few lysines of the abovementioned CRL substrate receptors subunits are also exposed to solvent. We performed the solvent accessibility analysis precisely the same as for the structural lysine desert search (see STAR Methods for details) based on the AlphaFold2 models of selected lysine-poor CRLs substrate receptors subunits, as their experimental structures, except for VHL (von Hippel-Lindau disease tumor suppressor), remain unsolved. Interestingly, not all their lysines are solvent accessible, making them even less prone to lysine-dependent ubiquitination, with the most extreme case of SOCS1 (suppressor of cytokine signaling 1), which decreased its lysines’ content 15-fold and possesses only one but buried lysine (Figure 5B; Table S13). VHL complex-free monomer has all its lysines exposed (Figure 5B), but when it associates with the elongin B, elongin C, and cullin 2, access to one or two lysines may be restricted (Figure 5C), likely making them inaccessible for any modifications. Moreover, the burial of lysines within the complex might be exploited by other CRL substrate receptors; therefore, their lysine solvent accessibility could differ from the one calculated for their models of complex-free monomers. This may indicate an evolutionary pressure to limit the ubiquitination of these CRL substrate receptors.

### Lysine-scarce SOCS1 and VHL exhibit reduced susceptibility to ubiquitination by cullin-RING ligases, yet remain prone to ubiquitination per se

To investigate the vulnerability of CRLs substrate receptors that have evolved lysine deserts through "lysine desertification" to ubiquitination by their respective ligase complexes, we measured the ubiquitination of SOCS1 and VHL, as well as their all-lysine-deficient variants (VHL K159R, K171R, K196R; SOCS1 K118R), in HEK293 cells. Our approach involved monitoring their ubiquitination responses to the inhibition of neddylation, a process critical for the functional activity of CRLs,^34^ as well as to a blockade of E1 enzymes.

To this end, we utilized the NanoBRET technology (Promega), a quantitative system commonly used in studies on protein-protein interactions.^35^^,^^36^^,^^37^^,^^38^ Additionally, we performed an internal validation of the system, wherein an examination of ubiquitination levels was carried out across varying concentrations of NanoLuc-VHL and HaloTag-ubiquitin plasmids. Subsequently, the NanoBRET assay was employed, generating a signal that exhibits a proportional increase corresponding to the extent of ubiquitination. The data, as illustrated in Figure S4, affirm the system’s responsiveness to alterations in the plasmid concentrations, as well as its ability to discern competitive substrate ubiquitination in the presence of HA-ubiquitin and substantiate the system’s suitability for facilitating a quantitative investigation of ubiquitination levels.

**Figure 6:**
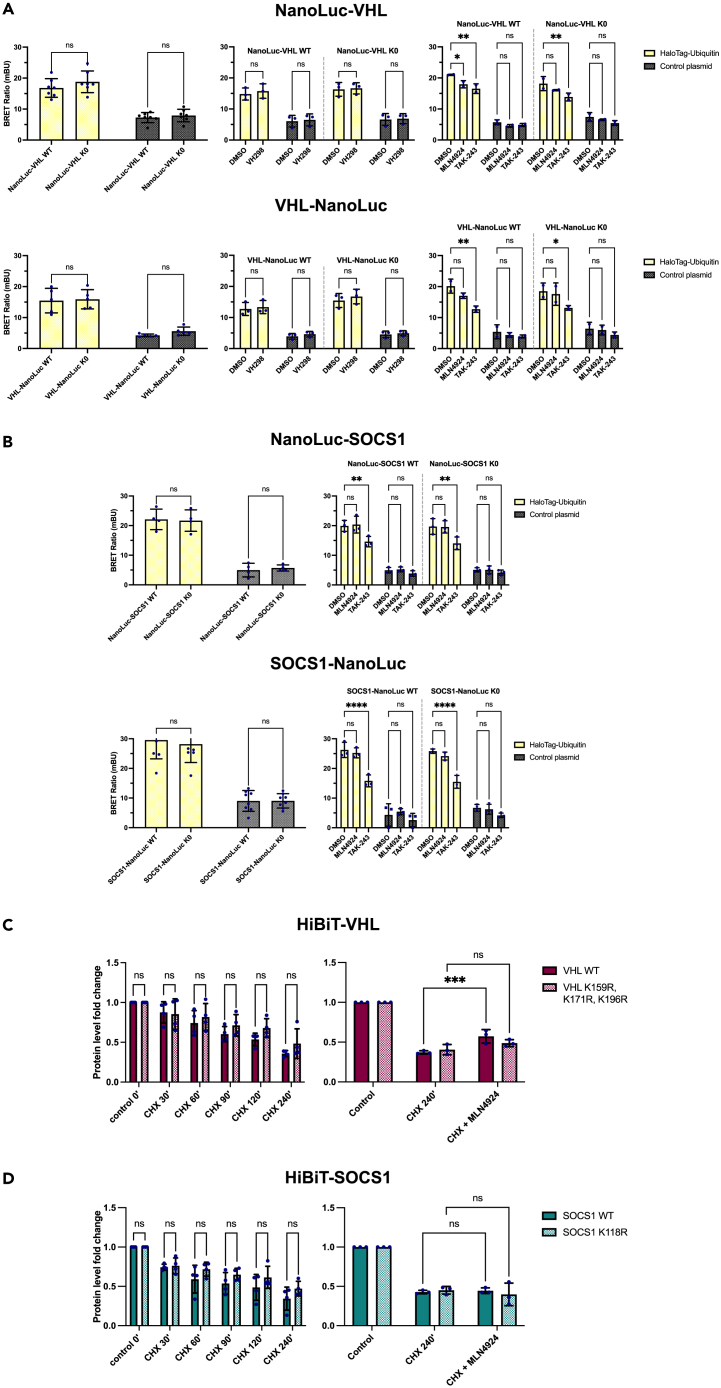
VHL and SOCS1 lysine deserts protect against CRL-mediated ubiquitination, but not against ubiquitination per se (A and B) VHL and SOCS1 wild-type (WT) and their lysine-less variants’ (K0) ubiquitination measured by NanoBRET assay with transient expression of the HaloTag-ubiquitin or the control plasmid pHTN HaloTag CMV-neo and NanoLuc-tagged (at its N-/C-terminus as indicated) protein of interest, as described in STAR Methods. Where specified, cells were also co-treated with 5 μM MLN4924 neddylation inhibitor for 5 h or 5 μM TAK-243 E1 inhibitor for 4 h; dimethyl sulfoxide (DMSO) served as a control. For VHL, further assessments were conducted by inhibiting its substrate-recognition domain using a 150 μM concentration of VH298 compound for a duration of 4 h, ensuring the VHL ubiquitination signal was not a result of substrate ubiquitination. Error bars denote the standard deviation from the mean derived from separate biological replicates (comparisons between WT vs. K0 without any additional inhibitor: eight for SOCS1-NanoLuc, seven for NanoLuc-VHL, five for VHL-NanoLuc and four for NanoLuc-SOCS1; three for VHL with VH298 and SOCS1 with MLN4924/TAK-243, two for VHL with MLN4924/TAK-243); dots represent the biological replicates. Each biological replicate is a mean of three technical replicates. Data was analyzed using two-way ANOVA and the significance levels obtained from the Šidák’s/Tukey’s multiple comparisons test are indicated for the compared conditions (Tukey’s test was used only to analyze data for VHL with MLN4924/TAK-243) (∗∗ - p ≤ 0.01; ∗ - p ≤ 0.05; ns - not significant). (C and D) VHL and SOCS1 wild-type (WT) and their lysine-less variants’ (K0) turnover measured by cycloheximide (CHX) assay with transient expression of the HiBiT-tagged protein of interest and treatment with 50 μg/mL CHX for the indicated time. In an independent set of experiments, cells were co-treated with 5 μM MLN4924 neddylation inhibitor. Protein levels were measured and normalized to the number of living cells as described in STAR Methods; assays were normalized to the corresponding measurement for each protein variant from control time 0’. Error bars denote the standard deviation from the mean derived from separate biological replicates (four in the VHL CHX chase assay and three in the SOCS1 CHX chase assay and the MLN4924 experiments); dots represent the biological replicates. Each biological replicate is a mean of three technical replicates. Data was analyzed using two-way ANOVA and the significance levels obtained from the Šidák’s/Tukey’s multiple comparisons test are indicated for the compared conditions (Tukey’s test was used only to analyze data for MLN4924) (∗∗∗ - p ≤ 0.001; ns - not significant).

Both the wild-type VHL and SOCS1, as well as their lysine-free variants, displayed similar levels of ubiquitination (Figures 6A and 6B), which suggests that the ubiquitination occurring on these proteins is independent of lysine residues. To eliminate the possibility that the observed ubiquitination signal arises from substrate ubiquitination rather than VHL itself, we treated cells with VH298, a compound known for its ability to selectively block the domain of VHL responsible for recognizing a majority of its substrates.^39^^,^^40^ Our analysis revealed no discernible differences between the untreated and treated conditions, as well as between the different protein variants (Figure 6A), supporting the notion that the observed ubiquitination stems from non-lysine VHL ubiquitination. Due to the absence of a selective inhibitor that specifically targets the SOCS1 binding surface, we were unable to perform analogous validations for this protein, however we deem it improbable that the detected ubiquitination signal originates from ubiquitinated substrates in both experimental cases of NanoLuc-labeled SOCS1 (at the N-/C-terminus, respectively).

**Figure 7:**
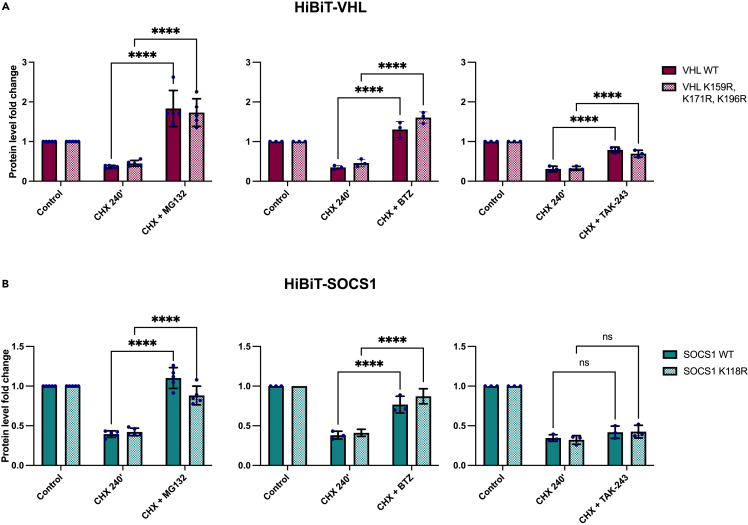
VHL and SOCS1 undergo proteasome-dependent degradation but differ in their ubiquitination function (A and B) VHL and SOCS1 wild-type (WT) and their lysine-less variants’ (K0) turnover measured by cycloheximide (CHX) assay with transient expression of the HiBiT-tagged protein of interest and co-treatment with 50 μg/mL CHX for 4 h and a specified compound - 20 μM MG132 proteasome inhibitor, 10 μM bortezomib (BTZ) proteasome inhibitor or 5 μM TAK-243 E1 inhibitor in a set of independent experiments. Protein levels were measured and normalized to the number of living cells as described in STAR Methods; assays were normalized to the corresponding measurement for each protein variant from control time 0’. Error bars denote the standard deviation from the mean derived from separate biological replicates (five in the experiments with MG132 and three in the experiments with BTZ and TAK-243); dots represent the biological replicates. Each biological replicate is a mean of three technical replicates. Data was analyzed using two-way ANOVA and the significance levels obtained from the Tukey’s multiple comparisons test are indicated for the compared conditions (∗∗∗∗ - p ≤ 0.0001; ns - not significant).

The inhibition of neddylation, achieved by subjecting the cells to MLN4924 treatment,^41^ did not lead to a significant reduction in the ubiquitination levels of any of the SOCS1 variants, as well as three out of the four VHL variants (with the exception being the NanoLuc-VHL wild-type). Conversely, the inhibition of E1 enzymes using TAK-243^42^ had a substantial negative impact on their ubiquitination levels (Figures 6A and 6B). These findings suggest that the predominant ubiquitination observed in SOCS1 and VHL is not a consequence of their involvement in CRL complexes.

Furthermore, to determine the turnover of SOCS1 and VHL and their lysine-free variants, also in response to MLN4924 neddylation inhibitor, we carried out cycloheximide assays (CHX). Here, we assessed the levels of SOCS1 and VHL variants tagged with High BiT (HiBiT, 11 amino acid tag; Promega) in HEK293 cells and detected their levels with a reagent containing the complementary peptide Large BiT (LgBiT; 17.6 kDa; Promega), which binds to HiBiT with high affinity generating luminescence. This provides quantitative protein abundance measurements in a linear dynamic range. Noteworthy, there is no risk of introducing additional ubiquitination sites when tagging proteins with HiBiT, as the HiBiT tag was validated as not prone to ubiquitination (information from the Promega R&D Department). During the CHX chase experiment (Figures 6C and 6D), we did not observe significant differences in the stability between the SOCS1 and VHL variants. However, concerning the influence of MLN4924, we observed no impact on the stability of either the wild-type or lysine-free SOCS1 variant (Figure 6D). In contrast, there was a moderate stabilization of the wild-type VHL, whereas its lysine-free variant remained unaffected (Figure 6C). Given VHL harbors three lysine residues, it is plausible that a lysine desert has evolved to restrain its ubiquitination and subsequent turnover during interactions within the CRL complex. This hypothesis is bolstered by the unaffected stability of the lysine-free variant under MLN4924 treatment. Conversely, SOCS1, with its single buried lysine creating a virtually total lysine desert, likely to resist CRL-mediated ubiquitination and avoid CRL-induced turnover.

### Contrasting turnover of lysine-deficient SOCS1 and VHL

Next, we aimed to determine the role of VHL and SOCS1 ubiquitination. To address this, we explored how inhibiting ubiquitination using an E1 inhibitor (TAK-243) or proteasome inhibitors (MG132 or bortezomib) would influence the degradation rates of both wild-type and lysine-free HiBiT-tagged SOCS1 and VHL, utilizing a comparable CHX assay. In all instances, the use of proteasome inhibitors resulted in a substantial accumulation of both SOCS1 and VHL variants, proving that both proteins undergo degradation that is dependent on the proteasome (Figures 7A and 7B). However, the inhibition of E1 enzymes did not significantly alter the stability of SOCS1 variants (Figure 7B), whereas it had a contrasting positive effect on VHL variants’ stability (Figure 7A).

These findings suggest different ubiquitination regulatory roles: VHL exhibits a partial reliance on this process for proteasomal degradation, whereas SOCS1 undergoes proteasomal degradation that is independent of ubiquitination. We corroborated this with an internal validation using BRD4 as a control to represent proteins degraded through both proteasome and ubiquitination pathways,^43^ while ornithine decarboxylase (ODC) served as a control for proteasome-dependent but ubiquitination-independent degradation.^44^ As anticipated, our validation study revealed similar turnover patterns between SOCS1/ODC and VHL/BRD4 in response to proteasome and E1 inhibition (Figure S5), thereby affirming the reliability of our HiBiT-based system in detecting proteins regulated by both ubiquitination and the proteasome.

To assess the comprehensive influence of lysine desert on protein stability, we initiated arginine-to-lysine triple point mutations at three separate locations in the lysine deserts of both HiBiT-tagged VHL and SOCS1 proteins, examining their stability through the CHX assay. For VHL, the addition of more lysine residues invariably led to reduced stability, which was rescued in the presence of neddylation or E1 inhibitors (Figure S6A), demonstrating that its lysine desert’s function is to mitigate ubiquitination by neddylation-regulated ubiquitin ligases, most likely cullins. On the other hand, SOCS1 exhibited no noticeable change, implying a potential specific mechanism that involves the ubiquitination of its limited residues or a high resistance to ubiquitin-dependent degradation (Figure S6B).

### Lysine deserts do not directly correlate with human proteins half-life

Since our experiments demonstrated that lysine deserts do not reduce the possibility of proteasomal degradation, we investigated whether there is a correlation between the half-life and the presence of lysine deserts in cell-specific human proteomes. To this end, we obtained protein turnover datasets from two large-scale proteomic studies.^45^^,^^46^ The dataset prepared by Mathieson and colleagues provided information on the turnover of 3555–4653 human proteins in primary cells, namely B cells, NK cells, hepatocytes, and monocytes, while Li and colleagues measured half-lives of 1428–1904 short-lived human proteins in four cell lines: U2OS, HEK293T, HCT116, and RPE1 (see STAR Methods for a summary of the number of analyzed proteins in each cell type). We found no correlation between the length of the lysine desert and the length of protein half-life in any dataset, irrespective of whether we analyzed the absolute length of the lysine desert region, its proportion to the whole protein sequence, or we focused on the subset comprising the 10% shortest living proteins (Data S1). Combined with the ubiquitination and turnover studies on SOCS1 and VHL, these analyses indicate that lysine deserts not necessarily influence the half-life of the corresponding human proteins, potentially due to effective non-lysine regulation by the UPS.

## Discussion

Functional components of the UPS exposed to ubiquitination should be equipped with mechanisms to prevent accidental proteolytic destruction. Selective pressure to avoid lysine residues, especially in intrinsically disordered regions, may underpin a strategy to avoid redundant ubiquitination of ubiquitin-proteasome components. To determine this possibility, we designed and implemented a bioinformatic pipeline to quantitatively search for lysine-free regions and investigate their evolution and functional roles.

We noted that the abundance of lysine deserts in *Actinobacteria* is most prevalent among species that possess proteasomes and utilize a pupylation pathway. The Pup-proteasome system (PPS) plays a key role in mycobacterial stress responses.^47^ For example, nitrogen starvation or reactive nitrogen species secreted by host macrophages in response to infection by *M. tuberculosis* induce PPS.^47^^,^^48^ Perhaps the abundance of lysine deserts in the *M. tuberculosis* proteome promotes the feasible degradation of only specific nitrogen metabolic network components, such as the HrcA repressor of chaperonin, which promote the nitrite reductase NirBD to assimilate nitrogen from nitrate.^49^ Furthermore, we found that *Mycobacterium* phages’ sequences contain several to dozens of times more lysine-depleted proteins than phages of bacteria not equipped with the PPS. Possibly this contributes to limiting pupylation and degradation of phages’ proteins enabling more efficient infection or killing of the mycobacterial host exposed to stress conditions.

We performed a similar screen for the presence of proteins with a lysine desert among the proteomes of model eukaryotic organisms and identified many E3s, ubiquitin-adaptor proteins, and other components of the cellular proteostasis system containing long sequence stretches completely devoid of lysine. One example of the latter is the ubiquitin-like (UBL) domain of BAG6. Kampmeyer and colleagues showed that introducing lysine residues into the BAG6 lysine-free sequence leads to increased ubiquitination and proteasomal degradation driven by its associated partner, E3 RNF126,^17^ which itself is also an example of a protein with a highly conserved lysine desert. We observed that in human E3s, predominantly among the class of the RING ligases, lysine-depleted regions are present primarily in disordered regions (Figures S3D and S3E). Perhaps avoidance of lysine modification by ubiquitin in these parts is required for their localization, conformation, activity and substrate binding.^50^^,^^51^ In addition to the UPS-related proteins, we identified elements such as NF-Y and RNA exosome complex components with significant lysine-deficient regions. Specifically, only NFYA in NF-Y and two subunits in the RNA exosome complex - EXOSC4 and EXOSC6 - feature lysine deserts. This lysine scarcity may divert UPS targeting from these elements to other complex components, potentially facilitating proper subunit turnover and complex disassembly.

Interestingly, many lysine desert E3s aggregate their lysine remnants within the RING domain, which interacts with E2. This may indicate pressure for specific auto-ubiquitination in the ordered cluster zone but not in the disordered lysine-free region. The example of RNF12/RLIM, a 624 aa long E3 ligase with a large disordered lysine desert undergoing intensive auto-ubiquitination on the RING-localized lysine cluster while sparing the remaining part responsible for sorting and substrate binding, seems to support this hypothesis.^52^^,^^53^^,^^54^ Another example of highly conserved lysine desert proteins^17^ with lysine clusters are ubiquilins, which recognize ubiquitinated proteins and guide them to the proteasome.^55^ Ubiquilins position all of their lysines to the N-terminal ubiquitin-like domain (UBL), facilitating interaction with proteasome receptors, leaving the ubiquitin-associated domain (UBA), responsible for recognizing and binding ubiquitin on client proteins,^55^^,^^56^ completely lysine devoid. Therefore, ubiquilins likely do not expose lysines to the cytoplasmic environment when binding to the proteasome, presumably being protected against ubiquitination during functioning. Upon the substrate/partner dissociation, the protein may again become susceptible to ubiquitination. Thus, lysine clusters can be functional regulatory elements of lysine desert E3s and other proteostasis factors.

When searching for the most evolutionarily conserved lysine desert proteins, we noticed a gradient trend in the appearance of lysine deserts in higher eukaryotes and hypothesized that some proteins expand their lysine-depleted region as organisms evolve. The most prominent examples are substrate receptors of the CRLs such as VHL. *C. elegans* VHL-1 and *D. melanogaster* Vhl proteins' lysine-less region fraction is 26% and 31%, respectively, while in contrast, their orthologs in *M. musculus* and *H. sapiens* have lysine deserts stretching for 66% and 74% of their sequences, respectively, suggesting evolutionary pressure on lysine-free region elongation. Besides, the lysine desert region appears in VHL not as a result of sequence lengthening but due to the conversion of lysine present in simpler organisms (worm and zebrafish) to other amino acids (i.e., arginine) in mouse and human homologs. Notably, the lysine-less region in VHL covers the VHL substrate-binding domain.^57^

SOCS1, a CRL substrate receptor in humans and mice, features a single lysine, marking a significant reduction compared to its lower organism orthologs. Throughout its evolutionary progression from zebrafish to mammalian species, 15 lysines were predominantly substituted with arginines, which, although positively charged, are impervious to ubiquitin tagging. This divergence in lysine content could imply a distinct evolutionary pathway for zebrafish SOCS1, potentially driven by differing thermal environments, immune system demands, or ubiquitin-proteasome system dynamics between species, fostering unique regulatory avenues for protein stability and function. The role of SOCS1 in modulating cytokine signaling and immune responses^58^ necessitates meticulous control, which might be achieved through various mechanisms across species, leading to altered lysine configurations that suit specific functional and environmental prerequisites.

Furthermore, it is worth noting that the sole lysine found in both human and mouse SOCS1 is not solvent-accessible, which reduces the probability of its involvement in lysine-dependent ubiquitination. This is echoed in the context of VHL, where there is a notable decrease in the accessibility of its lysine sites when bound to elongin B, elongin C, and cullin 2 (Figure 5C), illuminating the potential role of evolutionary pressures in moderating the ubiquitination tendencies of CRL substrate receptors. Corroborating this hypothesis, our utilization of the neddylation inhibitor MLN4924 affirmed that lysine deserts can function as a “shield,” limiting ubiquitination instigated by their intrinsic CRLs. However, as depicted in Figures 6A and 6B, this limitation did not extend to non-lysine modifications driven by other E3 ligases, as evidenced by reduced ubiquitination by E1 inhibitor, TAK-243, over MLN4924. For VHL, this strategy could streamline its degradation when it fails to associate with CRL adaptor proteins elongin B and C, aligning with observations by Schoenfeld and team who noted enhanced VHL stability upon interaction with elongin B and C.^59^ Notably, there are conflicting findings regarding the influence of CRL components on VHL stability. Pozzebon and colleagues demonstrated that the expression of Gam1, an adenoviral protein, leads to VHL ubiquitination and degradation dependent on cullin 2 and cullin 5, but not elongin B and C.^60^ Our data indicate that the CRL complex may not directly oversee the ubiquitination of VHL and its lysine-lacking variant, underlining a potential discrepancy in CRL’s role in VHL ubiquitination.

Quantitative analysis revealed no major turnover rate differences between the wild-type and lysine-free variants of VHL and SOCS1. However, these results contradict Wu et al., who asserted a CUEDC2-regulated, ubiquitination-enhanced degradation process for SOCS1.^61^ Yet, their experimental approach, which makes the analysis of non-lysine modification virtually impossible, indicates extensive polyubiquitination of SOCS1, despite only one lysine in this protein. Moreover, SOCS1 pull-down approach used in this study did not exclude the scenario that these modifications involve substrates bound by SOCS1. Thus, these outcomes do not invalidate our conclusions derived from methods demonstrating the potential for lysine-deficient proteins to experience non-lysine ubiquitination. Supporting this assumption, recent Trim-Away assay results showed that the TRIM21 E3 efficiently degrades lysine-less substrates, potentially in tandem with a non-lysine ubiquitination mechanism.^62^ This suggests that UPS may recruit specialized E3s that control the abundance of lysine-deficient proteins. Moreover, midnolin was recently shown to function as an alternative degradation pathway that recruits substrates to the proteasome in an ubiquitin-independent way.^63^ Notably, among the 508 identified proteins that experienced substantial destabilization as a result of midnolin overexpression, 13% of them exhibited lysine-desert region (either at least 150 amino acids or constituting at least 50% of their sequence) (as listed in Table S7). This observation implies that lysine-desert proteins, akin to SOCS1, might employ proteasome-dependent turnover mechanisms that do not rely on ubiquitination.

In the course of delineating the function of lysine deserts in preserving the stability of E3 ligases VHL and SOCS1, we undertook investigations utilizing arginine-to-lysine mutations within the lysine desert regions of these proteins. Our hypothesis, predicting protection from proteasomal degradation by lysine deserts, was confirmed in the case of VHL, where additional lysine residues contributed to lower stability. This instability is most likely due to modification by cullins, with which VHL forms a complex, as it is rescued at a similar level by the inhibition of neddylation, E1 enzymes, or the proteasome itself. SOCS1, conversely, demonstrated an inherent resilience, unyielding to the augmented lysine environment and maintaining a stable profile. Notably, SOCS1 contains a greater number of potentially non-lysine ubiquitination sites compared to VHL, as illustrated in Figure S7A. This observation could elucidate why SOCS1 experiences substantial ubiquitination on these non-lysine residues and why the introduction of lysines to disrupt its lysine desert does not significantly affect its turnover, as it suggests that the ubiquitination of SOCS1 serves purposes other than degradation.

We also did not observe a correlation between the extent of lysine deserts and protein half-life in human protein turnover datasets from proteomics studies (Data S1). This suggests that a lysine desert does not necessarily increase overall protein stability; rather, it can either provide relative protection against degradation (as observed in arginine-to-lysine VHL variants) or simply reduce ubiquitination, as seen in the case of the proteasome substrate shuttle RAD23A,^17^ where lysine desert protects against ubiquitination but does not affect proteasomal degradation. This raises the intriguing question of whether the UPS can employ non-lysine ubiquitination or proteasomal degradation pathways that operate independently of ubiquitination to regulate proteins within the lysine-deficient proteome. Furthermore, we discerned the following trend: proteins with extensive lysine deserts and higher frequency of serine and threonine amino acids — prone to non-lysine ubiquitination — generally possess reduced half-lives, especially within the proteomes of human monocytes and hepatocytes (Figure S7B). This observation highlights a complex regulatory role for lysine deserts and these specific amino acids in dictating protein stability, unveiling a promising avenue for more focused studies to further elucidate this intricate relationship.

### Limitations of the study

In this study, while we have made strides in uncovering the potential roles and prevalence of lysine deserts in proteostasis, several limitations persist. First, despite identifying possible functions of lysine deserts in averting untimely ubiquitin-dependent proteolysis, there remains a gap in understanding the full scope of lysine-free regions in proteins, including the potential roles in stabilizing molecular interactions, or limiting non-ubiquitin modifications of the lysine. Furthermore, we were unable to completely elucidate the pathways involved in the turnover of the lysine-deficient proteome, not ruling out the potential involvement of autophagy or cellular extrusion pathways that rely on extracellular vesicles. Our analysis of the ubiquitination of VHL and SOCS1 did not discern the exact ubiquitin ligases mediating this process or identify the specific sites of non-lysine ubiquitination. Moreover, the regulatory role of non-lysine ubiquitination of lysine-depleted proteins remains unproven, maintaining a level of ambiguity regarding its competition with other post-translational modifications. Addressing these issues will necessitate further investigations employing a diverse array of experimental approaches to grasp the multidimensional implications of lysine deserts in UPS and beyond. Future studies should venture into a more detailed scrutiny of non-lysine ubiquitination pathways and their biological repercussions, to render a holistic comprehension of the complexity in proteostasis.

## STAR★Methods

### Key resources table

**Table:** 

REAGENT or RESOURCE	SOURCE	IDENTIFIER
**Chemicals, peptides, and recombinant proteins**		

Bortezomib	Merck	Cat#504314
Cycloheximide	BioShop	Cat#CYC003.5
Dicoumarol	Merck	Cat#287897
FuGENE HD Transfection Reagent	Promega	Cat#E2312
MG132	Selleck Chemicals	Cat#S2619
MLN4924	Selleck Chemicals	Cat#S7109
MZ1 PROTAC	Tocris	Cat#6154
TAK-243	Selleck Chemicals	Cat#S8341
VH298	Selleck Chemicals	Cat#S8449

**Critical commercial assays**		

Nano-Glo HiBiT Lytic Detection Assay	Promega	Cat#N3040
CellTiter-Fluor Cell Viability Assay	Promega	Cat#G6080
NanoBRET Nano-Glo Detection Systems	Promega	Cat#N1661
CellTiter-Glo 2.0 Cell Viability Assay	Promega	Cat#G9241

**Deposited data**		

https://github.com/n-szulc/lysine_deserts	This work	https://doi.org/10.5281/zenodo.7545561

**Experimental models: Cell lines**		

Human: Flp-In HEK293	ThermoFisher Scientific	Cat#R75007

**Oligonucleotides**		

Primers for VHL, SOCS1, ODC, and BRD4 cloning and mutagenesis - see Table S19	This work	N/A

**Recombinant DNA**		

HiBiT-SOCS1 WT	Azenta Life Sciences	N/A
HiBiT- and NanoLuc-tagged constructs, see Table 6	This work	N/A
HA-ubiquitin	Addgene	Cat#18712
HaloTag-ubiquitin	Promega	Cat#N2721
NanoLuc-BRD4	Promega	Cat#N1691
pHTN HaloTag CMV-neo	Promega	Cat#G7721
pBiT3.1-N	Promega	Cat#N2361
pNLF1-N	Promega	Cat#N1351
pNLF1-C	Promega	Cat#N1361

**Software and algorithms**		

cd-hit web server	http://cd-hit.org	N/A
DSSP (v. 3.0.0) used within the Biopython python module	https://anaconda.org/salilab/dssp	N/A
GraphPad Prism 9 (v. 9.5.0)	https://www.graphpad.com	N/A
IUPred3 standalone	https://iupred3.elte.hu	N/A
Magellan Pro	https://lifesciences.tecan.com/software-magellan	N/A
Own codes	https://github.com/n-szulc/lysine_deserts	N/A
REVIGO web server	http://revigo.irb.hr	N/A
TMHMM-2.0 standalone	https://services.healthtech.dtu.dk/service.php?TMHMM-2.0	N/A
UniProt Retrieve/ID mapping	https://www.uniprot.org/id-mapping	N/A
WebGestalt web server	http://www.webgestalt.org	N/A

### Resource availability

#### Lead contact

• Further information and requests for resources and reagents should be directed to and will be fulfilled by the lead contact, Wojciech Pokrzywa (wpokrzywa@iimcb.gov.pl).

#### Materials availability

• Plasmids generated in this study are available on request.

#### Data and code availability

• Raw luminescence and fluorescence measurements from the CHX and NanoBret assays have been deposited at github.com/n-szulc/lysine_deserts and are publicly available as of the date of publication. DOI is listed in the key resources table.
• All original code has been deposited at github.com/n-szulc/lysine_deserts and is publicly available as of the date of publication. DOI is listed in the key resources table.
• Any additional information required to reanalyze the data reported in this paper is available from the lead contact upon request.

### Experimental model and study participant details

#### Cell culture

Flp-In 293 HEK293 cell line (female embryonic kidney epithelial cell line; ThermoFisher Scientific, product no. 510021, lot no. 2348919; authentication certificate issued by the manufacturer - checked for viability, mycoplasma, sterility and β-galactosidase activity) were cultivated in Dulbecco’s Modified Eagle’s Medium (DMEM; D6429, Sigma) supplemented with 10% heat-inactivated Fetal Bovine Serum (FBS; F9665, Sigma) and 1% Antibiotic-Antimycotic (15240062, Gibco) at 37°C, 5% CO_2_ in a humidified incubator.

### Method details

#### Lysine deserts in bacteria and eukaryotes

All bacteria and eukaryotic reference proteomes (for 8881 and 1329 taxons, respectively) were downloaded from the UniProt database^19^ (from the FTP repository; data obtained on 25.05.2022; see Table S1 for the summary of downloaded data). In all performed analyses, sequences <150 aa were excluded. For selected taxons, namely, *M. tuberculosis* H37Rv (virulent), *M. smegmatis*, *C. glutamicum*, *S. ceolicolor*, *L. ferrooxidans*, *B. subtilis*, *E. coli*, *S. cerevisiae*, *C. elegans*, *D. melanogaster*, *M. musculus*, and *H. sapiens*, number of transmembrane helices (TMH) for each sequence in their proteome were predicted using TMHMM-2.0 software.^20^^,^^21^ For analyses concerning these taxons, proteins with a predicted number of TMH >2 were excluded. Summary of a number of sequences prior and after filtering can be found in Tables S14 and S15. Sequences with a lysine desert of a declared type (lysine-less region of min. 150 aa or constituting min. 50% of the sequence) were counted in each proteome and a fraction of sequences with a given lysine desert was reported for each taxon or averaged for proteomes of the same bacteria class (only classes with at least 10 taxons were considered).

#### Lysine deserts in pupylomes

The dataset of pupylated proteins of *M. tuberculosis*, *M. smegmatis,* and *C. glutamicum* was downloaded from the PupDB database^23^ (data obtained on 04.08.2022). As some of the UniProt IDs in the obtained dataset were obsolete and could not be mapped directly to the UniProt reference proteomes of selected taxons, the UniProt Retrieve/ID mapping tool^19^ was used to retrieve the correct UniProt IDs. Among pupylated and non-pupylated proteins of the aforementioned taxons, sequences <150 aa and with >2 predicted TMH were excluded from further analyses (all proteins from the UniProt reference proteome of given taxon were considered as non-pupylated except those reported in the PupDB). The remaining proteins were screened for the presence of a lysine desert.

#### Lysine deserts in phages

Proteomes of *Mycobacterium*, *Escherichia* and *Bacillus* phages were downloaded from the UniProt database using the mycobacterium AND (taxonomy_id:10239), escherichia AND (taxonomy_id:10239), and bacillus AND (taxonomy_id:10239) queries, respectively (data obtained on 31.07.2022). Proteomes marked as outliers as well as those with <40 sequences were excluded from further analyses (see the summary of proteomes selected for analysis in Table S1). Next, for each phages’ group separately, all sequences, excluding those <150 aa, were concatenated into one fasta file and clustered using the cd-hit web server^64^ (with the default parameters, identity cutoff = 0.9) to create non-redundant pan proteome (summary of pan proteomes’ properties is presented in Table S16). Sequences with a lysine desert of declared type were counted in each pan proteome and a fraction of sequences with a given lysine desert was reported.

#### Lysine deserts in Orthologous Groups

##### Dataset

Lysine desert search among the Orthologous Groups (OGs) of *Saccharomycetaceae*, *Rhabditida*, *Drosophilidae*, *Rodentia*, and *Hominidae* was performed based on the datasets from the eggNOG5 5.0 database^24^ (data obtained on 31.07.2022).

##### Data preprocessing

For each mentioned taxonomic family/order, we selected Multiple Sequence Alignment (MSA) files of their OGs, which fulfilled the following conditions: (i) the presence of minimum four sequences, (ii) the presence of a minimum of 60% of the taxonomic family’s/order’s taxons (each taxonomic family/order from the eggNOG5 database owned a maximum number of taxons for which MSA files were constructed, however not all MSA files cover all taxons), (iii) median protein length of min. 150 aa, (iv) mean a number of predicted TMHs using the TMHMM-2.0 software ≤2, (v) the presence of at least one sequence from the representative organism for the particular taxonomic family/order - representative organisms are: *S. cerevisiae*, *C. elegans*, *D. melanogaster*, *M. musculus*, and *H. sapiens*. This data filtering resulted in the analysis of 50–68% of all available OGs (see Table S17).

##### Definition of a conserved lysine desert

For the purpose of OG-based analyses, we broadened the definition of the lysine desert, so the OG is considered as containing a conserved lysine desert if (i) at least 60% of its sequences possess a lysine desert region of a minimum length of 150 aa, or (ii) at least 60% of its sequences possess a lysine desert region constituting a minimum of 50% of OG’s median sequence length.

##### Finding analogous OGs between different families/orders

The eggNOG5 database does not provide direct information on which OGs of different taxonomic ranks cover analogous proteins. Therefore, such “vectors” of corresponding MSAs from analogous OGs were derived based on *Opisthokonta* - a broad eukaryotic supergroup, including animals and fungi. Briefly, over 100.000 MSAs of OGs belonging to *Opisthokonta*, also obtained from the eggNOG5 database, were screened to find sequences of analogous proteins from *S. cerevisiae*, *C. elegans*, *D. melanogaster*, *M. musculus*, and *H. sapiens* and match them with their presence in OGs of *Saccharomycetaceae*, *Rhabditida*, *Drosophilidae*, *Rodentia*, and *Hominidae.* The analyses were not performed directly on the OGs from *Opisthokonta*, as although their MSAs cover all aforementioned model organisms, they also usually contain long gaps regions (due to coverage of multiple evolutionary distant organisms) which would not allow for comparison lysine desert conservation between selected families/orders. The IDs of corresponding MSAs of OGs of *Saccharomycetaceae*, *Rhabditida*, *Drosophilidae*, *Rodentia*, and *Hominidae* are available at https://github.com/n-szulc/lysine_deserts.

#### Disorder predictions

The probability of each residue being disordered was calculated for all sequences in the human proteome, excluding those <150 aa and with the predicted number of TMH >2. Disorder predictions were obtained using two approaches: (i) by running the standalone version of the IUPred3^26^ software with the default parameters, (ii) by obtaining the pLDDT values from the AlphaFold2 models^28^ of the human proteome downloaded from the AlphaFold Protein Structure Database^29^ (proteome ID: UP000005640_9606_HUMAN_v3; since AlphaFold2 models of proteins >2700 aa are split to separate overlapping files, such proteins were also excluded from the analysis).

#### Structural lysine desert algorithm

##### Dataset

The structural lysine desert screen was performed on human proteome downloaded from the AlphaFold Protein Structure Database (proteome ID: UP000005640_9606_HUMAN_v3; since AlphaFold2 models of proteins >2700 aa are split to separate files, such proteins were also excluded from the analysis).

##### Data preprocessing

Each protein structure was parsed using the Biopython^65^ python module (v. 1.79) and list of contacts (neighbors) (default contact cut-off = 4.0 Å; defined as the shortest euclidean distance between any heavy atoms of the two residues) together with solvent-accessible surface area (SASA) and relative solvent accessibility (RSA) values obtained using the DSSP program^66^ (v. 3.0.0); Sander method^67^ was used for SASA normalization to acquire RSA) were calculated for each residue.

##### Aim

The algorithm intends to find the longest, uninterrupted lysine-less regions among solvent-exposed residues that remain in contact. Therefore, buried residues may also break the continuity of the structural lysine desert, as there may be no exposed neighbors to spread to (cases visible in Figure 3G when the entire protein is lysine-less yet the structural lysine desert does not equal 100%).

##### Algorithm

The complete code and documentation, including the algorithm’s visualization, are available at https://github.com/n-szulc/lysine_deserts. Briefly, all contacts are analyzed for each residue that is not a lysine and is solvent-exposed (RSA cut-off value > 0.2). If there is no solvent-exposed lysine among these contacts, the exposed contacts are added to the temporary list containing residues of the structural lysine desert; otherwise, the algorithm stops. Next, all contacts of the aforementioned contacts are analyzed. If there is no solvent-exposed lysine among them, the exposed ones are added to the temporary list containing residues of the structural lysine desert. The algorithm stops if such exposed ones are absent or a solvent-exposed lysine occurs. The algorithm repeats and saves the three most extended structural lysine deserts.

##### Remarks

When iterating over contacts, if a residue has no calculated SASA or RSA value due to the DSSP error, it is omitted, and the algorithm proceeds to the next one, except for lysine. In such a case, lysine is always considered solvent-exposed.

#### Length of lysine desert and half-life

The protein half-lives datasets were obtained from recent high-throughput proteomic studies.^45^^,^^46^ The dataset from Mathieson and colleagues provided information on the turnover of human proteins in primary cells - B cells, NK cells, hepatocytes, and monocytes, while the dataset from Li and colleagues outlined half-lives of short-lived human proteins measured in four cell lines - U2OS, HEK293T, HCT116, and RPE1. Mathieson and colleagues usually reported half-life values from two replicates for a given type of primary cells, mean protein half-life was calculated in all such cases; Li and colleagues reported only one value of the protein half-life in a given cell line. Gene symbols reported by Mathieson and colleagues and UniProt IDs provided by Li and colleagues were mapped to the UniProt reference human proteome to associate half-life values with representative proteins; half-life values whose identifier was unmapped were discarded from further analyses. Longest lysine-less region was calculated in all human proteins, excluding those <150 aa, with predicted (using the TMHMM-2.0 software) number of TMH >2, and with outlier half-life values (from 0.01 and 0.99 quantiles) for the whole datasets analyses or with half-life values within the 0.1 quantile, and expressed as either a nominal value or sequence fraction. A summary of the number of analyzed proteins with assigned half-life values is presented in Table S18.

#### Over-representation analysis

The GO-based over-representation analyses of human lysine desert min. 150 aa/min. 50% proteins, regardless of and along with lysine clusters, were performed using the WebGestalt web server with default parameters^68^; our filtered human proteome served as background. The false discovery rate (FDR) was controlled to 0.05 using the Benjamini-Hochberg method for multiple testing. The results were visualized as treemaps using the modified R scripts generated by the REVIGO web server^69^ (species specified as *Homo sapiens*, the rest of parameters was set as default).

#### Plasmid construction

The sequence and ligation independent cloning (SLIC) method,^70^ was used to construct HiBiT and NanoLuc fusion vectors. HiBiT fusion vectors were constructed using the parental vector pBiT3.1-N (N2361, Promega). To generate the HiBiT-VHL vector, the pBiT3.1-N vector was linearized with SacI, and HindIII enzymes, dephosphorylated and mixed with human VHL sequence PCR-amplified from HEK293 cDNA (VHL protein sequence correspondonding to UniProt ID: P40337). An identical procedure, with the exception of vector linearization performed using BamHI enzyme, was carried out to generate HiBiT-ODC (ODC protein sequence corresponding to UniProt ID: P11926) and HiBiT-BRD4, although the BRD4 sequence was cloned from the NanoLuc-BRD4 vector (N1691; Promega). Synthesis of HiBiT-SOCS1 was ordered from Azenta Life Sciences (SOCS1 protein sequence correspondonding to UniProt ID: O15524). To obtain HiBiT-SOCS1 K118R, R56K R65K R69K, R170K R172K R181K, R188K R193K R201K and HiBiT-VHL K159R K171R K196R, R64K R69K R113K, R79K R107K R108K, R176K R177K R182K variants, splice-PCRs were performed using HiBiT-SOCS1 and HiBiT-VHL vectors as templates, respectively. NanoLuc fusion vectors were constructed using the parental vectors pNLF1-N (N1351, Promega) and pNLF1-C (N1361, Promega) to obtain N-terminal and C-terminal NanoLuc-tagged protein fusions, respectively. To generate NanoLuc-VHL and NanoLuc-SOCS1 vectors, the pNLF1-N vectors were linearized with XbaI and XhoI enzymes, dephosphorylated, and linearized vectors were mixed with VHL or SOCS1 sequences, which were PCR-amplified using HiBiT-VHL or HiBiT-SOCS1 vectors as templates. For VHL and SOCS1 cloning into pNLF1-C vectors, the vector was linearized with PvuI and XhoI enzymes. The list of primers’ sequences used to generate the constructs is available in Table S19.

#### NanoBRET ubiquitination assay

##### Cells preparation

HEK293 cells were seeded in 6-well plates at 800.000 cells per well. After 6–8 hours, cells were transiently transfected with 1 or 2 μg (or 1/0.1/0.01 μg in the system validation setup presented in Figure S4) HaloTag-ubiquitin (N2721, Promega) or the control vector pHTN HaloTag CMV-neo (G7721, Promega) and 0.01 or 0.02 μg (or 0.01 μg in the system validation setup) NanoLuc-tagged SOCS1, VHL expression constructs (acceptor to donor ratio was maintained at the ratio of 100:1, while in the system validation setup at the ratios: 100:1, 10:1 and 1:1); additionally, where indicated for the validation setup, with 0.9 μg HA-ubiquitin (18712, Addgene). Transfection was carried out using the FuGENE HD Transfection Reagent (E2312, Promega) according to the manufacturer’s guidelines, in the Opti-MEM I Reduced Serum Medium, no phenol red (11058021, Gibco), maintaining the 2:1 FuGENE HD:DNA ratio. After 20–22 hours, cells were trypsinized, counted, and resuspended in the Opti-MEM containing 4% FBS and 1% Antibiotic-Antimycotic at a concentration of 200.000 cells/ml.

##### NanoBret assay

The bioluminescence resonance energy transfer (BRET) ubiquitination assay was performed using the NanoBRET Nano-Glo Detection Systems (Standard) (N1661, Promega) according to the manufacturer’s guidelines. Briefly, the HaloTag 618 ligand was added to the cells (at 100 nM concentration), and 100 μl of the cell suspension was plated per well of white 96-well tissue culture plates (655083, Greiner). Dimethyl sulfoxide (DMSO) was added (1 μl/ml) instead of the HaloTag 618 ligand to the control cells. For 20-22 hours, cells were treated with 5 μM MLN4924 (S7109, SelleckChemicals), 5 μM TAK-243 (S8341, SelleckChemicals) or DMSO (for vehicle control) for 5 hours. For the compound preparation, 10 mM of the MLN4924 and 5mM of the TAK-243 stock solutions in DMSO were diluted in the Opti-MEM to 100 μM concentration (20x), and 5 μl of the solution was added to appropriate wells. After the treatment, the NanoBRET Nano-Glo Substrate diluted in Opti-MEM was added to wells, and donor and acceptor light emissions were immediately measured using the TECAN Infinite 200 Pro plate reader equipped with the Magellan Pro software with the Blue2 NB and the Red NB filters, and integration time of 1000 ms. BRET ratios in mBRET units were calculated by dividing the acceptor emission (Red NB) by the donor luminescence (Blue2 NB) and multiplied by 1000. The values were corrected by subtracting the BRET ratio measured in equivalent samples that received DMSO instead of the HaloTag 618 ligand.

##### Cell viability assay

To assess cell viability during the NanoBret assays, they were multiplexed with the CellTiter-Glo 2.0 Cell Viability Assay (G9241, Promega) following the manufacturer’s guidelines. Briefly, an equal volume (125 μl) of the CellTiter-Glo 2.0 Reagent was added to each well and mixed on a plate shaker for 5 minutes. After a 30-minute incubation, luminescence was measured using the TECAN Infinite 200 Pro plate reader equipped with the Magellan Pro software and an integration time of 1000 ms. The untransfected cells were used as the global viability reference.

#### Cycloheximide assay

##### Cells preparation

HEK293 cells were seeded in white 96-well tissue culture plates (655083, Greiner) at a density of 10.000 cells in a total volume of 100 μl per well. After 38–40 hours, cells were transiently transfected with 2.5 ng HiBiT-tagged VHL, SOCS1, ODC or 0.05 ng BRD4 expression constructs, diluted in carrier DNA (E4881, Promega) to obtain a final DNA amount of 50 ng/well. Transfection was carried out using the FuGENE HD Transfection Reagent (E2312, Promega) according to the manufacturer’s guidelines in the Opti-MEM I Reduced-Serum Medium (31985047, Gibco), maintaining the 3:1 FuGENE HD: DNA ratio. Cells were incubated for 23 hours at 37°C, 5% CO_2_.

##### Cycloheximide assay

Cycloheximide (CHX; CYC003.5, BioShop) was added to wells at a final 50 μg/mL concentration for up to 4 hours. Treatment was performed in triplicate. Where indicated, cells were concomitantly treated with 5 μM MLN4924 (S7109, Selleck Chemicals) for 5 hours, 5 μM TAK-243 (S8341, Selleck Chemicals) for 4 hours, 20 μM MG132 (S2619, Selleck Chemicals) for 4 hours or 10 μM bortezomib (BTZ; 504314, Merck) for 4 hours. Cells expressing HiBiT-BRD4 and HiBiT-ODC were additionally treated, where indicated, with 1 μM MZ1 PROTAC (6154, Tocris) for 4 hours and 150 μM dicoumarol (287897, Merck) for 2 hours, respectively. MLN4924 and dicumarol, the latter only in case of 60-minute CHX co-treatment, were added to the wells 1 hour before the first CHX dose. After a 4-hour incubation with CHX, the Nano-Glo HiBiT Lytic Detection Assay (N3040, Promega) was performed according to the manufacturer’s guidelines. Briefly, 120 μL of the Nano-Glo HiBiT Lytic Reagent was added to each well. Samples were mixed on an orbital shaker for 5 minutes and incubated for 10 minutes before luminescence measurement using the TECAN Infinite 200 Pro plate reader equipped with the Magellan Pro software and an integration time of 1000 ms. The luminescence measurements were normalized in each well to the number of living cells (see below).

##### Cell viability assay

To assess cell viability during the CHX assays, they were multiplexed with the CellTiter-Fluor Cell Viability Assay (G6080, Promega) following the manufacturer’s guidelines. Briefly, after 3-hour incubation with CHX, 20 μL of the 5x concentrated CellTiter-Fluor Reagent was added to all wells. Cells were incubated at 37°C for 1 hour, and fluorescence was measured using the Tecan Infinity M1000 fluorescence plate reader equipped with the Magellan Pro software with the parameters setup of 390nm_Ex_/505nm_Em_. The untransfected cells were used as the global viability reference.

#### Plotting

Phylogenetic trees were plotted using the ETE python toolkit^71^ (v. 3.1.2). Results of the CHX and NanoBret assays were visualized in the GraphPad Prism 9 (v. 9.5.0). All other plots were generated using the matplotlib (v. 3.5.1) and seaborn (v. 0.11.2) python modules. Graphics were created with BioRender.com.

### Quantification and statistical analysis

Experimental data are shown as mean and standard deviation and individual biological and technical replicates are indicated in each figure’s legend when applicable. Statistical analyses concerning lysine desert correlation with protein half-life were carried out using the SciPy^72^ (v. 1.7.3) python module and the results of the CHX and NanoBret assays were analyzed in the GraphPad Prism 9 (v. 9.5.0). Statistical tests included two-way ANOVA with post-hoc Šidák’s/Tukey’s/Dunnett’s multiple comparisons tests, Mann-Whitney U rank test, unpaired t-tests with Welch’s correction and Spearman rank-order correlation coefficient, as indicated in each figure’s legend where applicable. p values <0.05 were considered as significant.

## Supplemental information

Document S2. Tables S1 and S7–S11

Data S1. Analysis of lysine deserts’ correlation with protein half-life, related to Figure 7
