# Supplementary material for: Lysine deserts and cullin-RING ligase receptors: Navigating untrodden paths in proteostasis
Source: iScience. 2023 Oct 28;26(11):108344. doi: 10.1016/j.isci.2023.108344 (PMC10665810; doi:10.1016/j.isci.2023.108344)
Supplement: Document S1. Figures S1–S7 and Tables S2–S6 and S12–S19 [file mmc1.pdf]

iScience, Volume *26*

Supplemental information

Lysine deserts and cullin-RING ligase receptors: Navigating untrodden paths in proteostasis

Natalia A. Szulc, Małgorzata Piechota, Lilla Biriczová, Pankaj Thapa, and Wojciech Pokrzywa

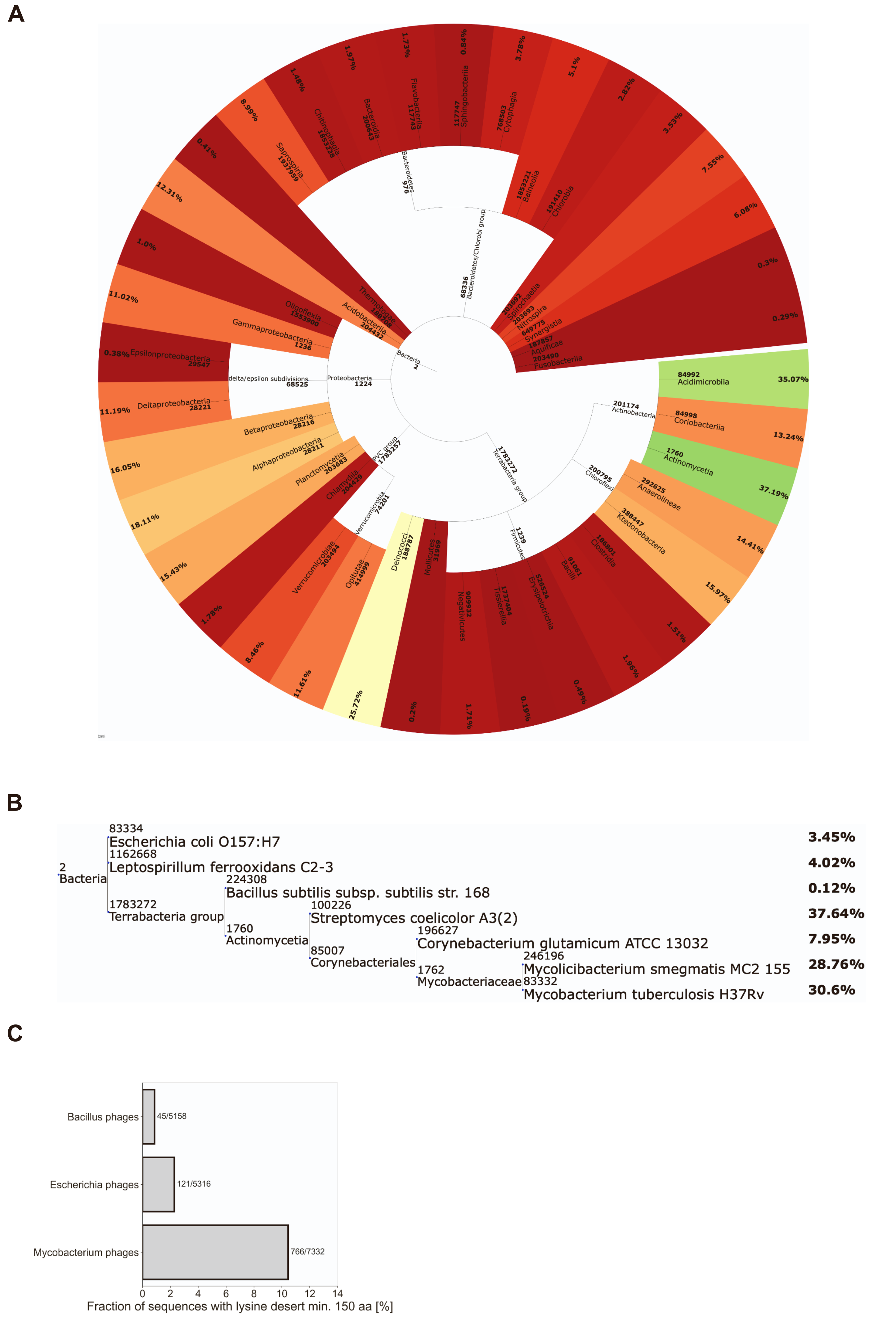

SFigure 1. Lysine deserts in bacteria are most prevalent in Actinobacteria and their phages, related to Figure 2. (A) Phylogenetic tree of different bacteria classes with a calculated average protein fraction with lysine desert min. 150 aa in the proteomes of their member taxons. Only classes with at least 10 member taxons were considered. The color gradient corresponds to the min-max normalization where red denotes classes with the lowest average fraction of desert min. 150 aa and green with the highest. **(B)** Phylogenetic tree of selected bacteria taxons from distinct classes with a calculated fraction of proteins with lysine desert min. 150 aa in their proteomes. **(C)** Bar plot of fractions of lysine desert min. 150 aa in pan proteome of selected phages' groups. The number of sequences with lysine desert and the total number of analyzed sequences are indicated to the right of each bar.

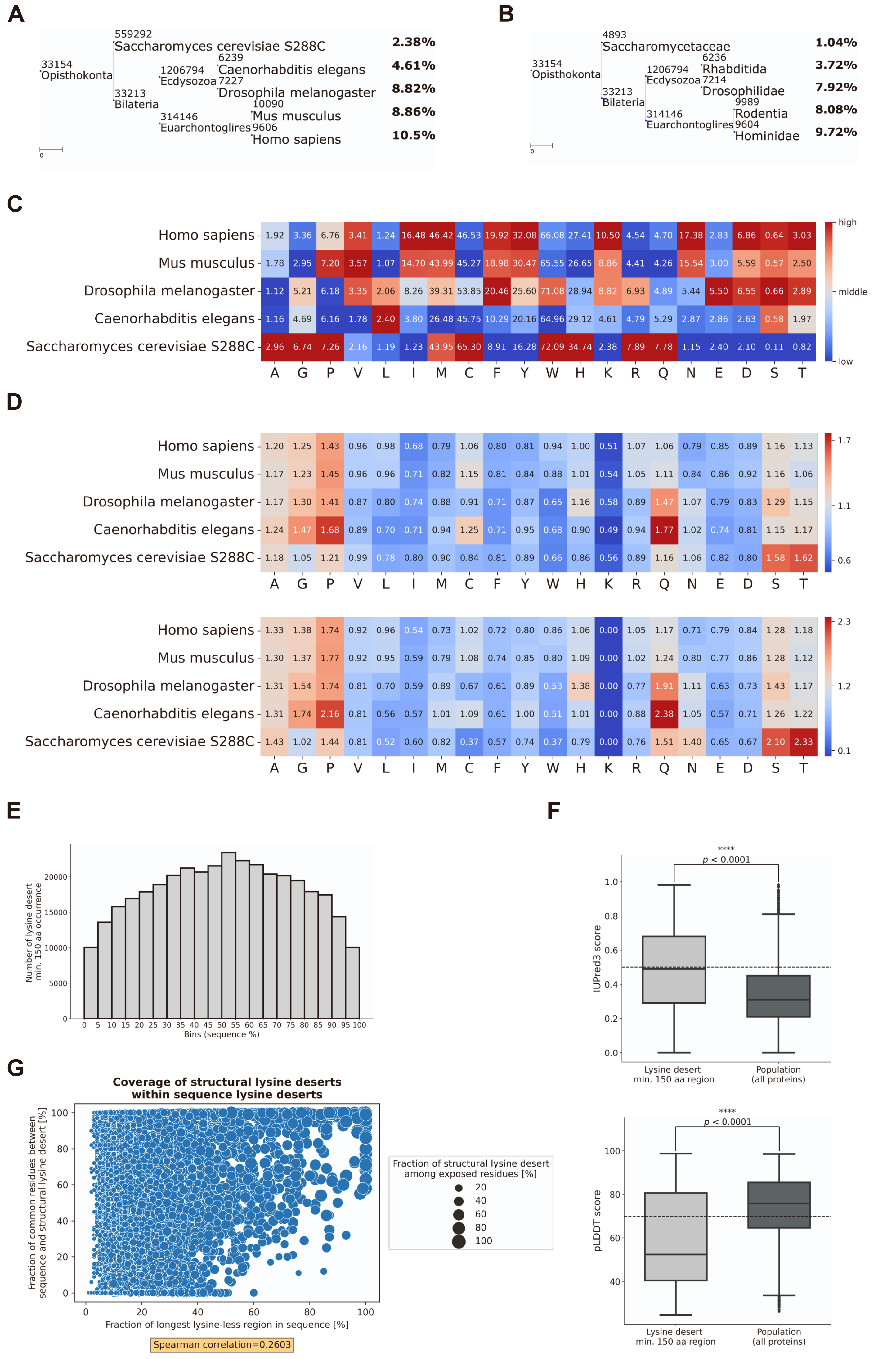

SFigure 2. Lysine deserts in eukaryotes ascend with growing organismal complexity, related to Figure 3. (A) Phylogenetic tree of selected eukaryotes taxons with a calculated fraction of proteins with lysine desert min. 150 aa in their proteomes. **(B)** Phylogenetic tree of selected taxonomic families/order, including model organisms used in the previous analysis, with calculated fractions of conserved lysine desert min. 150 aa among their OGs. **(C)** Heatmap of fractions of proteins with desert region min. 150 aa of each of 20 aa among proteomes of selected eukaryotic model organisms. **(D)** Heatmaps of relative fractions of each amino acid in sequences of selected eukaryotic model organisms with a lysine desert min. 150 aa normalized to the entire appropriate proteome; value of 1.00 indicates no change. Up - considering whole sequences; down - considering only the lysine desert regions. **(E)** Histogram of distribution of sequence location of lysine desert min. 150 aa regions in the human proteome. **(F)** Box plots of predicted disorder score of residues constituting lysine desert region min. 150 aa only vs. residues of all proteins in the human proteome. Up - sequence-based disorder predictions obtained using the IUPred3 software; higher values indicate a higher disorder probability. Down - structure-based disorder predictions based on the pLDDT values obtained for the AlphaFold2 models of the human proteome; lower values indicate a higher probability of disorder. Disorder cut-offs proposed by the method's authors are marked with dashed lines. The stars denote the significance levels per two-tailed p-value obtained from the Mann-Whitney U rank test. **(G)** Scatter plot showing coverage of residues building structural lysine deserts within residues constituting sequence lysine deserts in human proteome based on the AlphaFold2 models. The Spearman rank-order correlation coefficient is denoted below the plot.

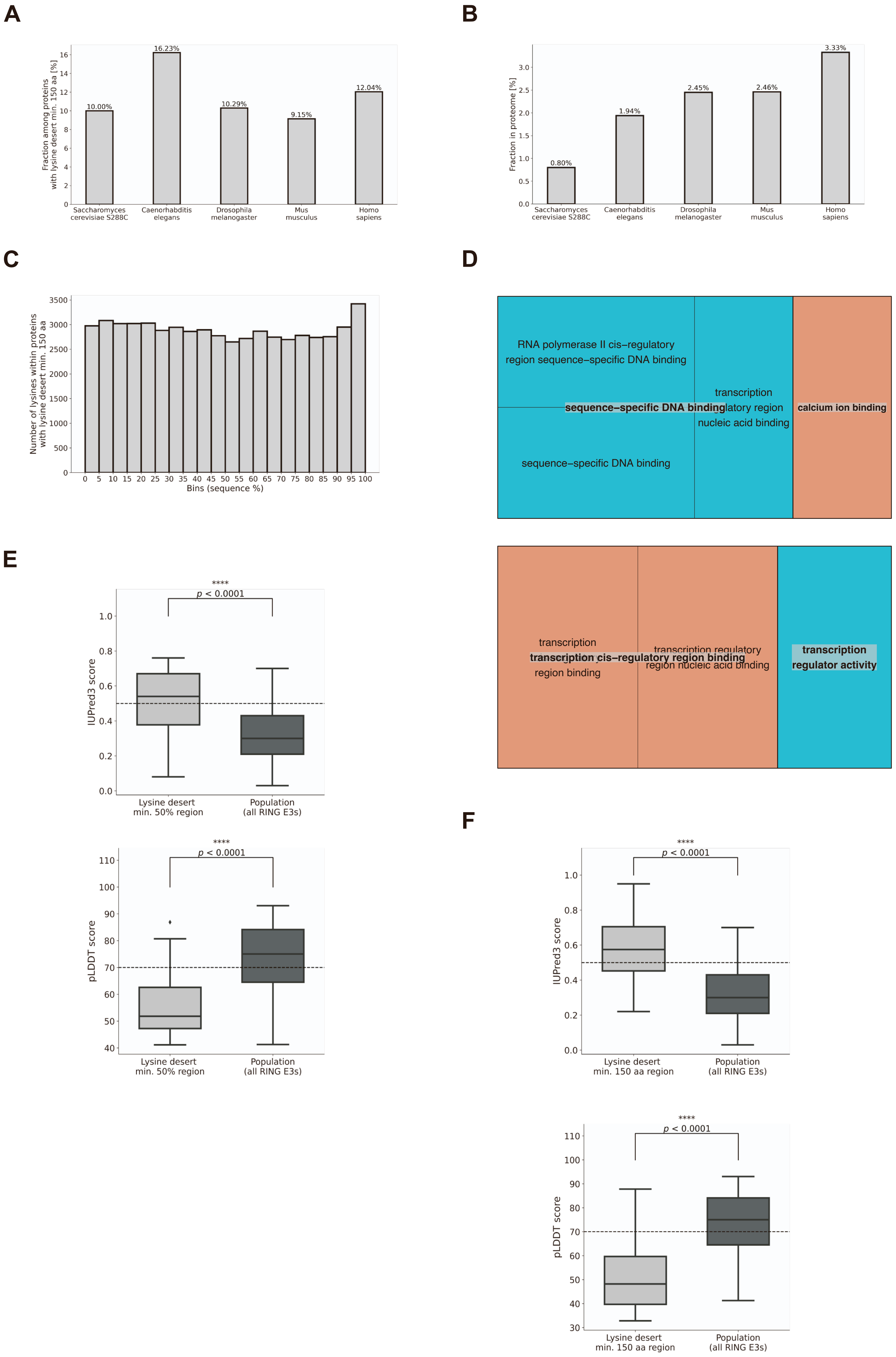

SFigure 3. Lysine deserts often co-occur with a lysine cluster, related to Figure 4. (A) Bar plot of the fraction of lysine clusters' occurrence among proteins with lysine desert min. 150 aa from selected model organisms. Exact values are denoted above each bar. **(B)** Bar plot of the fraction of lysine clusters' occurrence in proteomes of selected model organisms. Exact values are denoted above each bar. **(C)** Histogram of distribution of sequence location of lysines within proteins with lysine desert min. 150 aa from the human proteome. **(D)** GO molecular function terms found to be associated with proteins with lysine desert min. 150 aa (up) and with proteins with lysine desert min. 150 aa along co-occurring with a lysine cluster (down). **(E)** Box plots of predicted disorder score of residues constituting lysine desert regions min. 50% only in human RING E3s vs. residues of all human RING E3s. Up - sequence-based disorder predictions obtained using the IUPred3 software; higher values indicate a higher disorder probability. Down - structure-based disorder predictions based on the pLDDT values obtained from the AlphaFold2 models of the human proteome; lower values indicate a higher probability of disorder. Disorder cut-offs proposed by the authors of the methods are marked with dashed lines. The stars denote the significance levels per two-tailed p-value obtained from the Mann-Whitney U rank test. **(F)** Box plots of predicted disorder score of residues constituting lysine desert region min. 150 aa only in human RING E3s vs. residues of all in human RING E3s. Up - sequence-based disorder predictions obtained using the IUPred3 software; higher values indicate a higher disorder probability. Down - structure-based disorder predictions based on the pLDDT values obtained for the AlphaFold2 models of the human proteome; lower values indicate a higher probability of disorder. Disorder cut-offs proposed by the method's authors are marked with dashed lines. The stars denote the significance levels per two-tailed p-value obtained from the Mann-Whitney U rank test.

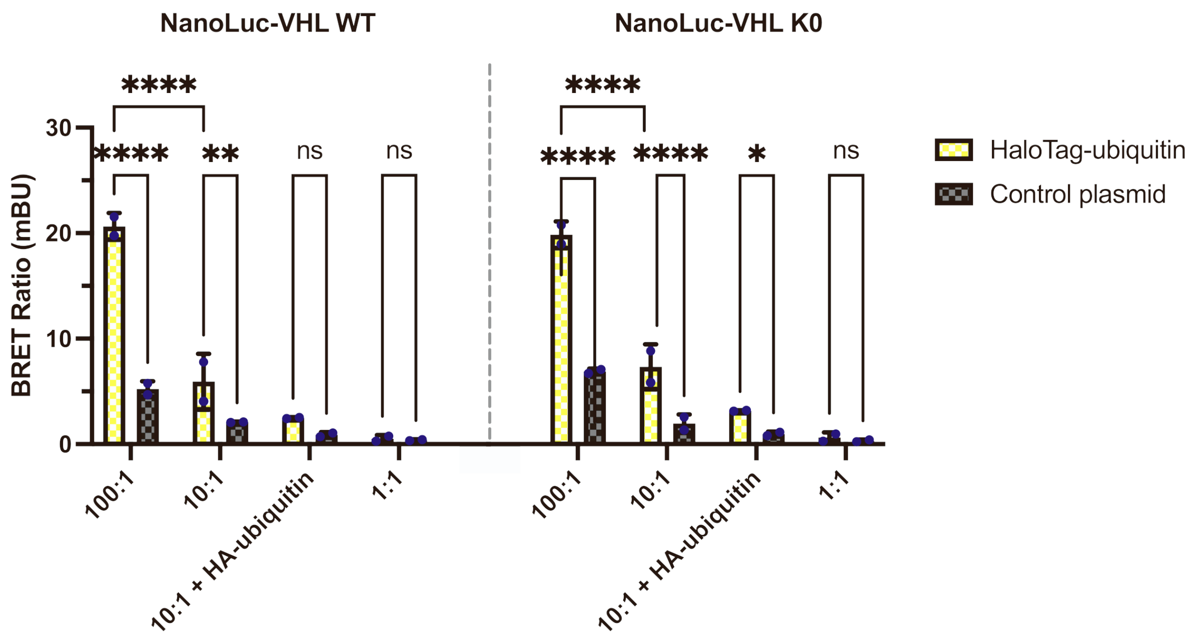

Acceptor	Acceptor [µg]	NanoLuc-VHL WT/K0 donor [µg]*	Acceptor to donor ratio	HA-ubiquitin [µg]	DNA carrier [µg]
HaloTag-ubiquitin	1	0.01	100:1	0	0.99
HaloTag-ubiquitin	0.1	0.01	10:1	0	1.89
HaloTag-ubiquitin	0.1	0.01	10:1	0.9	0.99
HaloTag-ubiquitin	0.01	0.01	1:1	0	1.98
Control plasmid	1.136	0.01	100:1	0	0.99
Control plasmid	0.114	0.01	10:1	0	1.89
Control plasmid	0.114	0.01	10:1	0.9	0.99
Control plasmid	0.01136	0.01	1:1	0	1.98

* Identical quantities in a given condition for both NanoLuc-VHL WT and NanoLuc-VHL K0 donors

SFigure 4. Establishing the quantitative sensitivity of the NanoBRET ubiquitination assay, related to Figure 6. Ubiquitination measured by NanoBRET assay with transient expression of 0.01 µg donor NanoLuc-VHL wild-type (WT) and lysine-less (K0) variants and the varying amounts of acceptors: HaloTag-ubiquitin or control plasmid pHTN HaloTag CMV-neo, as well as HA-ubiquitin; donor to acceptor ratios are indicated on the X axis following more details provided in the table. Error bars denote the standard deviation from the mean derived from two separate biological replicates; dots represent the biological replicates. Each biological replicate is a mean of three technical replicates. Data was analyzed using two-way ANOVA and the significance levels obtained from the Tukey's multiple comparisons test are indicated for the compared conditions (**** - $p \leq 0.0001$; ** - $p \leq 0.01$; * - $p \leq 0.05$; ns - not significant).

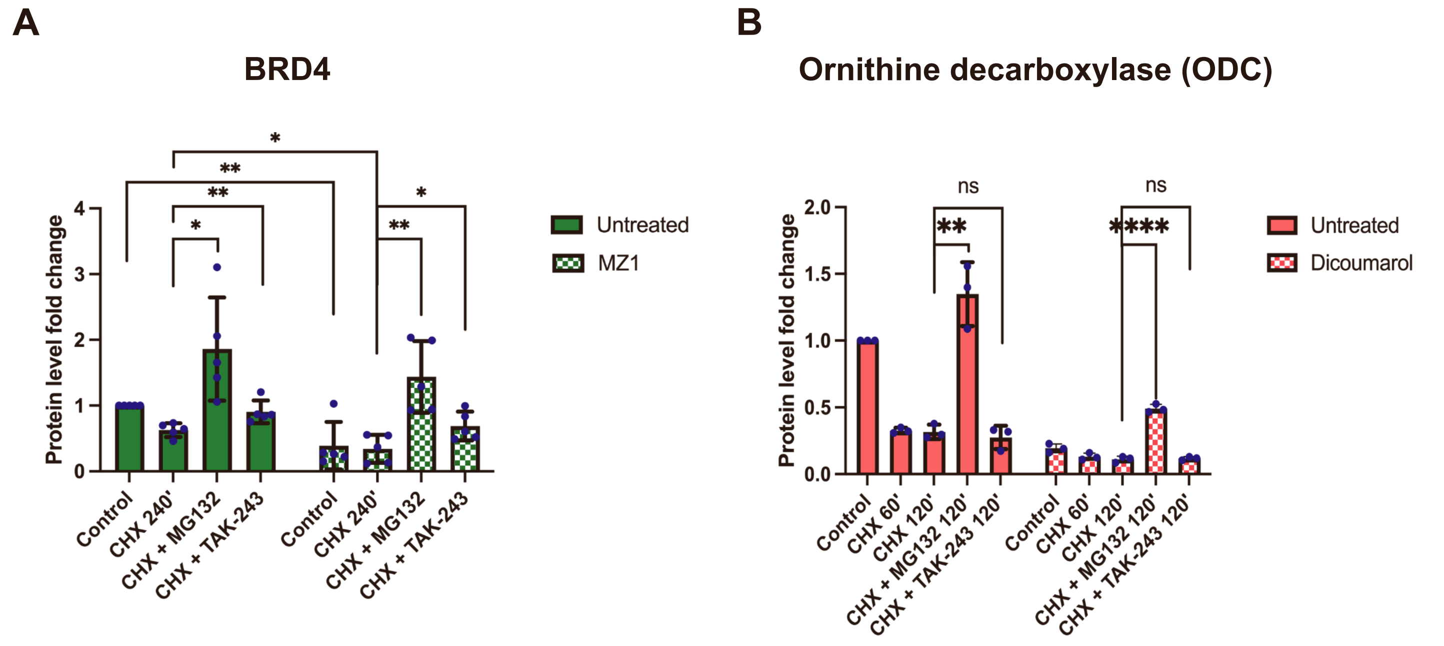

SFigure 5. Comparison of BRD4 and ornithine decarboxylase (ODC) turnover in response to proteasome and E1 inhibition, related to Figure 7. (A-B) The turnover of BRD4 and ODC was evaluated in a cycloheximide (CHX) assay utilizing transient expression of HiBiT-tagged proteins of interest and concurrent treatment with 50 µg/ml of CHX for the determined period. Where indicated, cells were co-treated with 20 µM MG132 proteasome inhibitor, 5 µM TAK-243 E1 inhibitor, 1 µM MZ1 PROTAC (only in the case of HiBiT-BRD4-expressing cells to induce its ubiquitination [S1]), or 150 µM dicoumarol, a NAD(P)H quinone oxidoreductase 1 inhibitor (only in the case of HiBiT-ODC-expressing cells to enhance ubiquitin-independent proteasomal degradation of ODC [S2]). Protein levels were measured and normalized to the number of living cells as described in STAR Methods; assays were normalized to the corresponding measurement for each protein variant from control time 0'. Error bars denote the standard deviation from the mean derived from separate biological replicates (five for BRD4 and three for ODC); dots represent the biological replicates. Each biological replicate is a mean of three technical replicates. The one-tailed significance levels obtained from unpaired t-tests with Welch's correction, conducted separately for each compared pair of conditions, are indicated on the plot (**** - p ≤ 0.0001; *** - p ≤ 0.001; ** - p ≤ 0.01; * - p ≤ 0.05; ns - not significant).

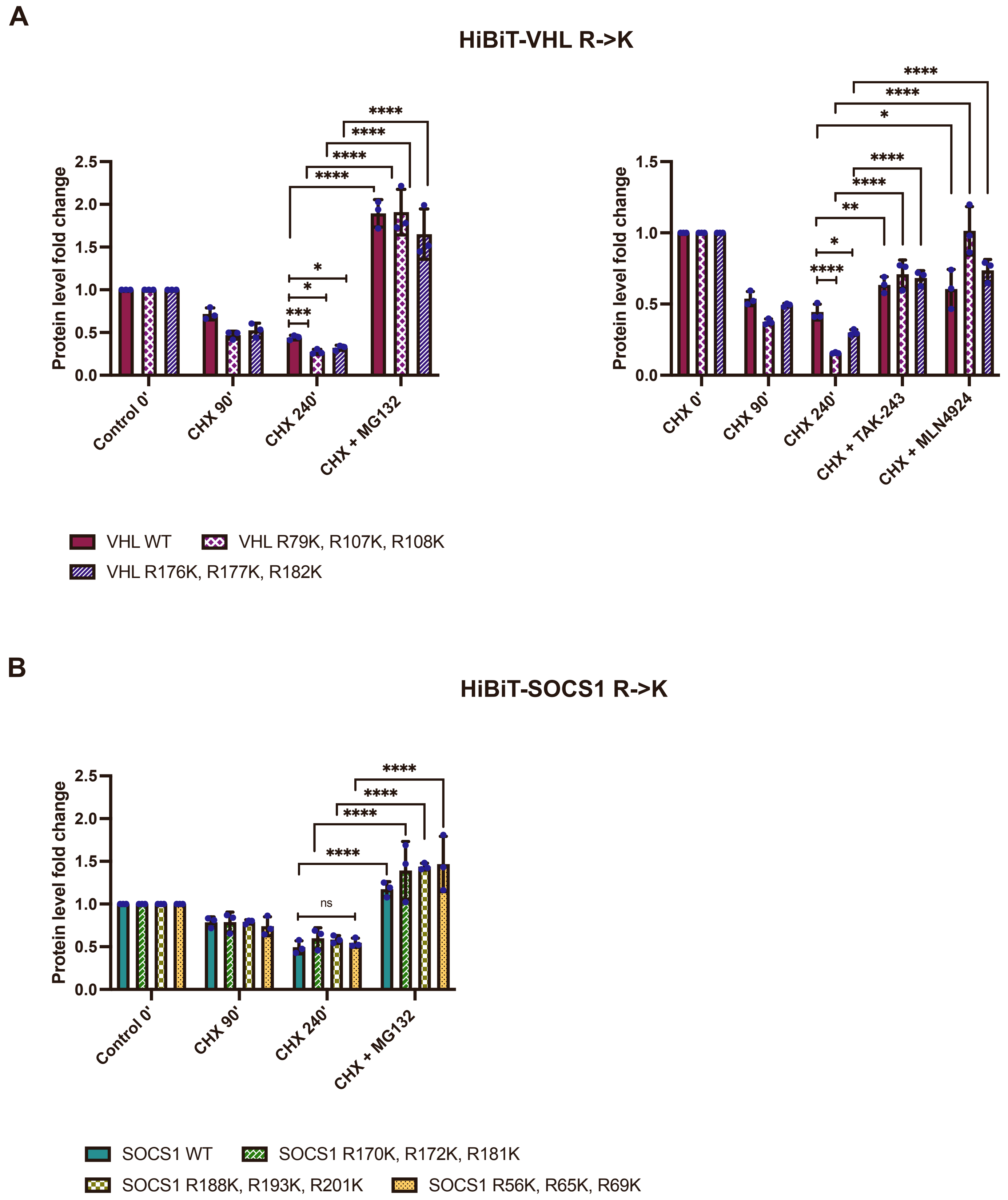

SFigure 6. Disruption of lysine deserts in VHL and SOCS1 leads to different consequences on their turnover, related to Figure 7. (A-B) VHL and SOCS1 wild-type (WT) and their lysine desert-disruptive (arginine to lysine; R->K) variants' turnover measured by cycloheximide (CHX) assay with transient expression of the HiBiT-tagged protein of interest and treatment with 50 µg/ml CHX for the indicated time. Where specified, cells were co-treated with 20 µM MG132 proteasome inhibitor for 4 hours, 5 µM MLN4924 neddylation inhibitor for 5 hours or 5 µM TAK-243 E1 inhibitor for 4 hours. Protein levels were measured and normalized to the number of living cells as described in STAR Methods; assays were normalized to the corresponding measurement for each protein variant from control time 0'. Error bars denote the standard deviation from the mean derived from three separate biological replicates; dots represent the biological replicates. Each biological replicate is a mean of three technical replicates. Data was analyzed using two-way ANOVA and the significance levels obtained from the Dunnett's multiple comparisons test are indicated for the compared conditions (**** - p ≤ 0.0001; *** - p ≤ 0.001; ** - p ≤ 0.01; * - p ≤ 0.05; ns - not significant).

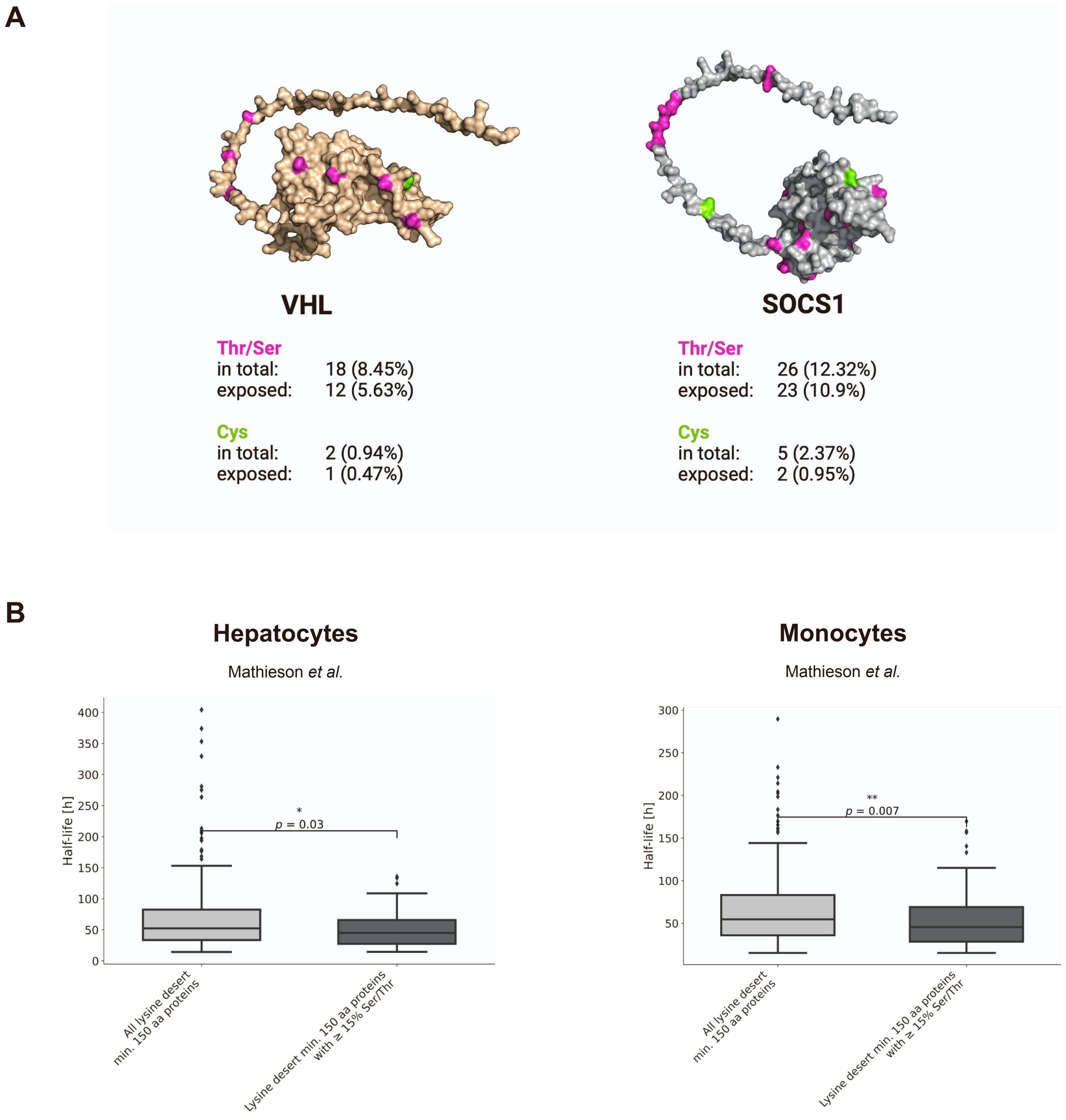

SFigure 7. Analysis of potential non-lysine ubiquitination sites in VHL and SOCS1 and the implications on proteome half-life in hepatocytes and monocytes, related to Figure 7. (A) Comparison between the number of serine/threonine (Ser/Thr) and cysteine (Cys) residues in VHL and SOCS1. Relative solvent accessibility (RSA) was calculated using the DSSP program and the Sander method based on AlphaFold2 models of VHL and SOCS1 (v4. models corresponding to UniProt IDs P40337 and O15524, respectively). Residues with RSA > 0.2 were considered as solvent-exposed. The AlphaFold2 models were visualized in the PyMOL software (Schrödinger) (v. 2.5.0). **(B)** Bar plots showing a comparison of half-lives between all lysine desert min. 150 aa human proteins and those rich in serine/threonines in hepatocytes and monocytes, based on a large-scale proteomic study [S3]. The stars denote the significance levels per two-tailed *p*-value obtained from the Mann-Whitney U rank test. Additional comparisons can be found in the https://github.com/n-szulc/lysine_deserts repository [S4].

Table S2. Summary of pupylomes of *M. tuberculosis*, *M. smegmatis,* and *C. glutamicum*, related to Figure 2.

	Number of proteins in proteome	Number of pupylated proteins	Fraction of pupylated proteins
M. tuberculosis	3995	54	1.35
M. smegmatis	6602	76	1.15
C. glutamicum	3093	43	1.39

Table S3. Summary of occurrence of lysine desert min. 50% in pupylomes of *M. tuberculosis*, *M. smegmatis*, and *C. glutamicum*, related to Figure 2.

	Total number of sequences	Total number of filtered[a] sequences	Average length of filtered[a] sequences [aa]	Number of sequences with lysine desert min. 50%	Fraction of sequences with lysine desert min. 50%
M. tuberculosis	54	49	405.86	4	8.16
M. smegmatis	76	66	406.83	1	1.52
C. glutamicum	43	39	370.23	1	2.56

[a] Excluding sequences <150 aa and with the predicted number of TMH >2.

Table S4. Summary of occurrence of lysine desert min. 150 aa in pupylomes of *M. tuberculosis, M. smegmatis*, and *C. glutamicum*, related to Figure 2.

	Total number of sequences	Total number of filtered[a] sequences	Average length of filtered[a] sequences [aa]	Number of sequences with lysine desert min. 150 aa	Fraction of sequences with lysine desert min. 150 aa
M. tuberculosis	54	49	405.86	8	16.33
M. smegmatis	76	66	406.83	2	3.03
C. glutamicum	43	39	370.23	1	2.56

[a] Excluding sequences <150 aa and with the predicted number of TMH >2.

Table S5. Summary of occurrence of lysine desert min. 50% in non-pupylated proteins of *M. tuberculosis*, *M. smegmatis*, and *C. glutamicum*, related to Figure 2.

	Total number of sequences	Total number of filtered[a] sequences	Average length of filtered[a] sequences [aa]	Number of sequences with lysine desert min. 50%	Percentage of sequences with lysine desert min. 50%
M. tuberculosis	3940	2729	386.62	710	26.02
M. smegmatis	6524	4601	355.82	1223	26.58
C. glutamicum	3050	1948	360.01	185	9.5

[a] Excluding sequences <150 aa and with the predicted number of TMH >2.

Table S6. Summary of occurrence of lysine desert min. 150 aa in non-pupylated proteins of *M. tuberculosis*, *M. smegmatis*, and *C. glutamicum*, related to Figure 2.

	Total number of sequences	Total number of filtered[a] sequences	Average length of filtered[a] sequences [aa]	Number of sequences with lysine desert min. 150 aa	Percentage of sequences with lysine desert min. 150 aa
M. tuberculosis	3940	2729	386.62	842	30.85
M. smegmatis	6524	4601	355.82	1341	29.15
C. glutamicum	3050	1948	360.01	157	8.06

[a] Excluding sequences <150 aa and with the predicted number of TMH >2.

Table S12. Orthologs of human E3 ligases with ≤ 5 lysines[a] in *D. rerio*, *X. silurana*, *G. gallus* and *M. musculus*, related to Figure 5.

D. rerio E3 ligase	*D. rerio* UniProt ID	*D. rerio* no of lysines	*X. silurana* E3 ligase	*X. silurana* UniProt ID	*X. silurana* no of lysines	*G. gallus* E3 ligase	*G. gallus* UniProt ID	*G. gallus* no of lysines	*M. musculus* E3 ligase	*M. musculus* UniProt ID	*M. musculus* no of lysines	*H. sapiens* E3 ligase	*H. sapiens* UniProt ID	*H. sapiens* no of lysines	CRL receptor
Kbtbd13a; Kbtbd13b	A0A140LGF5; X1WCK8	28; 20	Kbtbd13	F6U0R1	22	KBTBD13	A0A8V0Z2K6	14	Kbtbd13	Q8C828	5	KBTBD13	C9JR72	4	yes
Socs1A; Socs1B	Q6DEF9; E7FDH8	15;11	Socs1	Q5M8D9	15	SOCS1	B6RCQ2	13	Socs1	O35716	1	SOCS1	O15524	1	yes
Vhl; Vhll	A1L296; B3DHE4	5;7	Vhl	A9ULN6	6	VHL	A0A8V0Z8B2	6	Vhl	P40338	4	VHL	P40337	3	yes
-	-	-	-	-	-	-	-	-	Kctd11	Q8K485	2	KCTD11[b]	Q693B1	1	yes
Rnf126	A2RV40	6	Rnf126	Q6DIP3	3	RNF126	A0A1L1RZV7	4	Rnf126	Q91YL2	4	RNF126	Q9BV68	4	no
Fbxl15	H9KUW9	8	Fbxl15	Q5XGC0	16	FBXL15	F1NF36	11	Fbxl15	Q91W61	2	FBXL15	Q9H469	2	yes
Rnf181	Q7ZW78	8	Rnf181	Q5M974	8	-	-	-	Rnf181	Q9CY62	7	RNF181	Q9P0P0	5	no
Rnf6	F1R4P2	3	-	-	-	RNF6	A0A1D5NWA7	4	Rnf6	Q9DBU5	4	RNF6	Q9Y252	4	no
Rnf11a; Rnf11b	B8A662; B0V2S5	3;4	Rnf11	Q28H59	4	RNF11	F1NLF7	4	Rnf11	Q9QYK7	4	RNF11	Q9Y3C5	4	no
Rnf44	Q08CG8	5	Rnf44	A0A8J0PJJ6	6	RNF44	R4GIU9	5	Rnf44	Q8BI21	6	RNF44	Q7L0R7	5	no
-	-	-	Siah3 (Xenbase)	A0A6I8RL86	9	SIAH3	R4GKE1	7	Siah3	B2RWG3	5	SIAH3	Q8IW03	5	no
-	-	-	Fbxo27 (Xenbase)	Q0VA18	22	-	-	-	Fbxo27	Q6DIA9	8	FBXO27	Q8NI29	5	yes
Neurl2	A4IG40	16	Neurl2	F7CZK9	13	NEURL2	F1NIQ8	5	Neurl2	Q9D0S4	6	NEURL2	Q9BR09	5	yes
Rnf224	A5PMH4	11	-	-	-	-	-	-	Rnf224	Q3UIW8	3	RNF224	P0DH78	2	no

[a] Searched in the human proteome, excluding sequences <150 aa, with the predicted number of TMH >2, or annotated as membrane-bound.
[b] Although there are no known orthologs of KCTD11 from *D. rerio*, *X. silurana*, and *G. gallus*, we also consider it as decreasing its lysine content in the evolution, as its ortholog from *C. picta* turtle has 4 lysines (UniProt ID: A0A8C3H7N5).

Table S13. Relative solvent accessibility (RSA) values for lysines[a] of human E3 ligases with ≤ 5 lysines calculated using the AlphaFold2 models, related to Figure 5.

UniProtID	Gene symbol	AlphaFold2 model ID	Lysines indices	Lysines RSA
Q9H469	FBXL15	AF-Q9H469-F1-model_v3	180;268	0.63;0.76
Q8NI29	FBXO27	AF-Q8NI29-F1-model_v3	126;163;164;218;245	0.54;0.0;0.29;0.68;0.65
C9JR72	KBTBD13	AF-C9JR72-F1-model_v3	210;222;390;427	0.32;0.36;1.0;0.16
Q693B1	KCTD11	AF-Q693B1-F1-model_v3	31	0.79
Q9BR09	NEURL2	AF-Q9BR09-F1-model_v3	73;237;276;279;283	0.3;0.19;0.69;0.2;0.14
Q9Y3C5	RNF11	AF-Q9Y3C5-F1-model_v3	6;82;94;95	1.0;0.73;0.98;0.6
Q9BV68	RNF126	AF-Q9BV68-F1-model_v3	207;209;233;271	0.55;0.66;0.74;0.57
Q9P0P0	RNF181	AF-Q9P0P0-F1-model_v3	56;75;109;134;137	0.76;0.58;0.77;0.58;0.64
P0DH78	RNF224	AF-P0DH78-F1-model_v3	95;112	0.49;1.0
Q7L0R7	RNF44	AF-Q7L0R7-F1-model_v3	351;357;405;409;412	0.72;0.67;0.91;0.8;0.65
Q9Y252	RNF6	AF-Q9Y252-F1-model_v3	80;608;630;644	0.35;0.75;0.44;0.62
Q8IW03	SIAH3	AF-Q8IW03-F1-model_v3	23;25;45;173;213	0.97;0.7;0.91;0.07;0.5
O15524	SOCS1	AF-O15524-F1-model_v3	118	0.18
P40337	VHL	AF-P40337-F1-model_v3	159;171;196	0.53;0.67;0.41

[a] Searched in the human proteome, excluding sequences <150 aa, with the predicted number of TMH >2, or annotated as membrane-bound.

Table S14. Summary of selected bacteria proteomes for lysine desert analysis, related to STAR Methods.

	UniProt proteome ID	Total sequences in proteome	Fraction of filtered[a] sequences
M. tuberculosis **H37Rv**	UP000001584	3995	69.54
M. smegmatis **MC2 155**	UP000000757	6602	70.72
C. glutamicum **ATCC 13032**	UP000000582	3093	64.24
S. coelicolor **A3(2)**	UP000001973	8034	69.88
L. ferrooxidans **C2-3**	UP000007382	2413	65.98
E. coli **O157:H7**	UP000000558	5062	62.96
B. subtilis subsp. subtilis **str. 168**	UP000001570	4260	58.05

[a] Excluding sequences <150 aa and with the predicted number of TMH >2.

Table S15. Summary of selected eukaryotic proteomes, related to STAR Methods.

	UniProt proteome ID	Total sequences in proteome	Fraction of filtered[a] sequences
S. cerevisiae **S288C**	UP000002311	6059	76.35
C. elegans	UP000001940	19825	66.13
D. melanogaster	UP000000803	13823	75.69
M. musculus	UP000000589	21984	72.89
H. sapiens	UP000005640	20598	76.81

[a] Excluding sequences <150 aa and with the predicted number of TMH >2.

Table S16. Summary of proteomes of *Mycobacterium*, *Escherichia* and *Bacillus* phages, related to STAR Methods.

Phages group	Proteomes number	Filtered[a] proteomes number	Total sequence number in filtered[a] proteomes	Fraction of sequences from filtered[a] proteomes used for cd-hit	Total number of sequences in pan proteome[b]	Median sequence length in pan proteome[b]
Bacillus	149	133	18659	40.33	5158	256
Escherichia	263	245	22931	44.41	5316	260.5
Mycobacterium	527	527	53012	36.93	7332	271

[a] Excluding outlier proteomes and with <40 sequences.
[b] After running cd-hit.

Table S17. Summary of MSA files of OGs of *Saccharomycetaceae*, *Rhabditida*, *Drosophilidae*, *Rodentia*, and *Hominidae*, related to STAR Methods.

	Number of all MSA files	Number of filtered[a] MSA files	Fraction of filtered[a] MSA files	Median of median lengths of all MSA files	Median of median lengths of filtered[a] MSA files
Saccharomycetaceae	5524	3654	66.15	415.25	447.0
Rhabditida	18187	9123	50.16	337.0	398.0
Drosophilidae	13686	8638	63.12	404.0	463.0
Rodentia	21313	12714	59.65	414.0	483.0
Hominidae	20461	13988	68.36	407.0	478.0

[a] According to the procedure as described in this paragraph.

Table S18. Summary of the number of analyzed proteins with a measured half-life in each dataset, related to STAR Methods.

	**Number of proteins**	**Number of proteins mapped to the reference human proteome**	**Number of proteins used in the analysis[a] (whole dataset)**	**Number of proteins used in the analysis[b] (10% shortest living proteins only)**
**Mathieson *et al.*, 2018**				
B cells	4653	4134	3885	397
NK cells	3555	3203	3005	397
Hepatocytes	4242	3755	3342	342
Monocytes	4649	4193	3886	397
**Li *et al.*, 2021**				
U2OS	1428	1175	1140	117
HEK293T	1434	1151	1124	115
HCT116	1904	1571	1520	156
RPE1	1652	1335	1278	132

[a] Excluding sequences <150 aa, with the predicted number of TMH >2, and with outlier half-life values (from 0.01 and 0.99 quantile).
[b] Excluding sequences <150 aa, with the predicted number of TMH >2, and with half-life values equal or below 0.1 quantile.

Table S19. List of primers used in this study for cloning, related to STAR Methods. Nucleotides indicated by capital letters are additions to the primer, whereas those indicated by lowercase letters anneal to the template DNA.

Construct	Template	Primer sequences (forward and reverse)
HiBiT-VHL WT	HEK 293 cDNA	CGGCTGTTCAAGAAGATTAGCGGGAGCTCCatgccccggagggcggagaac<br>ATGCCTGCAGGTTTAAACCCCCGAAGCTTAatctcccatccgttgatgtg
NanoLuc-VHL WT	HiBiT-VHL WT	GCTGTGCGAACGCATTCTGGCGGGCTCGAGtatgccccggagggcggagaac<br>CCTGCAGGAATTGGGCCCAAATCTAGAttaatctcccatccgttgatgtg
VHL-NanoLuc WT	HiBiT-VHL WT	AATACGACTCACTATAGGGCTAGCAAAGCGatgccccggagggcggagaac<br>AAATCTTCGAGTGTGAAGACGCCGCTCGAatctcccatccgttgatgtg
HiBiT-VHL K159R K171R K196R	PCR I<br>template: HiBiT-VHL WT	CGGCTGTTCAAGAAGATTAGCGGGAGCTCCatgccccggagggcggagaac<br>gtaattctcaggcctgactaggctccggacaacctggaggcatcgctctctcagagtatacac
	PCR II<br>template: HiBiT-VHL WT	gtgtatactctgagagagcgatgcctccaggttgtccggagcctagtcaggcctgagaattac<br>ctccaggtctctctgcacatttgggtggtc
	PCR III<br>template: HiBiT-VHL WT	gaccacccaaatgtgcagagagacctggag<br>ATGCCTGCAGGTTTAAACCCCCGAAGCTTAatctcccatccgttgatgtg
	PCR IV<br>template: splice PCR I+II+III	CGGCTGTTCAAGAAGATTAGCGGGAGCTCCatgccccggagggcggagaac<br>ATGCCTGCAGGTTTAAACCCCCGAAGCTTAatctcccatccgttgatgtg
NanoLuc-VHL K159R K171R K196R	HiBiT-VHL K159R K171R K196R	GCTGTGCGAACGCATTCTGGCGGGCTCGAGtatgccccggagggcggagaac<br>CCTGCAGGAATTGGGCCCAAATCTAGAttaatctcccatccgttgatgtg
VHL-NanoLuc K159R K171R K196R	HiBiT-VHL K159R K171R K196R	AATACGACTCACTATAGGGCTAGCAAAGCGatgccccggagggcggagaac<br>AAATCTTCGAGTGTGAAGACGCCGCTCGAatctcccatccgttgatgtg
HiBiT-VHL R64K, R69K, R113K	PCR I<br>template: HiBiT-VHL WT	CGAGCGGTGGGAATTCTGGTGGAGGATCCatgccccggagggcggagaac<br>gggagggctcCTTcgagttcaccgaCTTcagcacgggc
	PCR II<br>template: HiBiT-VHL WT	gcccgtgctgAAGtcggtgaactcgAAGgagccctccc<br>caaaggtgaccCTTgtagctgtg

	PCR III<br>template: HiBiT-VHL WT	ccacagctacAAGggtcacctttg<br><br>AGATCTTCCGCTAGCTCCACCGGATCCTTAatctcccatccgttgatgtg
	PCR IV<br>template: splice PCR I+II+III	CGAGCGGTGGGAATTCTGGTGGAGGATCCatgccccggagggcggagaac<br><br>AGATCTTCCGCTAGCTCCACCGGATCCTTAatctcccatccgttgatgtg
HiBiT-VHL R79K R107K R108K	PCR I<br>template: HiBiT-VHL WT	CGAGCGGTGGGAATTCTGGTGGAGGATCCatgccccggagggcggagaac<br><br>cgcgcggactCTTattgcagaag
	PCR II<br>template: HiBiT-VHL WT	cttctgcaatAAGagtccgcgcg<br><br>gtagctgtggatCTTCTTgcccgtgccag
	PCR III<br>template: HiBiT-VHL WT	ctggcacgggcAAGAAGatccacagcta<br><br>AGATCTTCCGCTAGCTCCACCGGATCCTTAatctcccatccgttgatgtg
	PCR IV<br>template: splice PCR I+II+II	CGAGCGGTGGGAATTCTGGTGGAGGATCCatgccccggagggcggagaac<br><br>AGATCTTCCGCTAGCTCCACCGGATCCTTAatctcccatccgttgatgtg
HiBiT-VHL R176K R177K R182K	PCR I<br>template: HiBiT-VHL WT	CGAGCGGTGGGAATTCTGGTGGAGGATCCatgccccggagggcggagaac<br><br>cgtagagcgaCTTgacgatgtccagCTTCTTgtaattctcag
	PCR II<br>template: HiBiT-VHL WT	ctgagaattacAAGAAGctggacatcgtcAAGtcgctctacg<br><br>AGATCTTCCGCTAGCTCCACCGGATCCTTAatctcccatccgttgatgtg
	PCR III<br>template: splice PCR I+II	CGAGCGGTGGGAATTCTGGTGGAGGATCCatgccccggagggcggagaac<br><br>AGATCTTCCGCTAGCTCCACCGGATCCTTAatctcccatccgttgatgtg
NanoLuc-SOCS1 WT	HiBiT-SOCS1 WT	CTGGCGGCTGTGCGAACGCATTCTGGCGGGCTCGAGtatggtagcacacaatcaagtgg<br><br>AATCCTGCAGGAATTGGGCCCAAATCTAGAttaaatctggaaggggaagctgc
SOCS1-NanoLuc WT	HiBiT-SOCS1 WT	AATACGACTCACTATAGGGCTAGCAAAGCGatggtagcacacaatcaag<br><br>GAAATCTTCGAGTGTGAAGACGCCGCTCGAaatctggaaggggaagctgc
HiBiT-SOCS1 K118R	PCR I<br>template: HiBiT-SOCS1 WT	CGGCTGTTCAAGAAGATTAGCGGGAGCTCCatggtagcacacaatcaag<br><br>gccgctagccatcctcacgctcagggcg
	PCR II<br>template: HiBiT-SOCS1 WT	cgccctgagcgtgaggatggctagcggc<br><br>ATGCCTGCAGGTTTAAACCCCCGAAGCTTAaatctggaaggggaagctg

	PCR III<br><br>template: splice PCR I+II	CGGCTGTTCAAGAAGATTAGCGGGAGCTCCatggtagcacacaatcaag<br><br>ATGCCTGCAGGTTTAAACCCCCGAAGCTTAaatctggaaggggaagctg
NanoLuc-SOCS1 K118R	HiBiT-SOCS1 K118R	CTGGCGGCTGTGCGAACGCATTCTGGCGGGCTCGAGtatggtagcacacaatcaagtgg<br><br>AATCCTGCAGGAATTGGGCCCAAATCTAGAttaaatctggaaggggaagctgc
SOCS1-NanoLuc K118R	HiBiT-SOCS1 K118R	AATACGACTCACTATAGGGCTAGCAAAGCGatggtagcacacaatcaag<br><br>GAAATCTTCGAGTGTGAAGACGCCGCTCGAaatctggaaggggaagctgc
HiBiT-SOCS1 R56K R65K R69K	PCR I<br>template: HiBiT-SOCS1 WT	TCGAGCGGTGGGAATTCTGGTGGAGGATCCatggtagcacacaatcaag<br><br>gggcgcttgcCTTggtgattctCTTgtagtcggcgtggcttctgaaggtCTTgaagtgggtg
	PCR II<br>template: HiBiT-SOCS1 WT	cacccacttcAAGaccttcagaagccacgccgactacAAGagaatcaccAAGgcaagcgccc<br><br>AGATCTTCCGCTAGCTCCACCGGATCCTTAaatctggaaggggaagctgc
	PCR III<br>template: splice PCR I+II	TCGAGCGGTGGGAATTCTGGTGGAGGATCCatggtagcacacaatcaag<br><br>AGATCTTCCGCTAGCTCCACCGGATCCTTAaatctggaaggggaagctgc
HiBiT-SOCS1 R170K R172K R181K	PCR I<br>template: HiBiT-SOCS1 WT	TCGAGCGGTGGGAATTCTGGTGGAGGATCCatggtagcacacaatcaag<br><br>gtggccacgatCTTctgtctacacagctcttgcaggggCTTcacCTTtctctgtctc
	PCR II<br>template: HiBiT-SOCS1 WT	gagacagagaAAGgtgAAGcccctgcaagagctgtgtagacagAAGatcgtggccac<br><br>AGATCTTCCGCTAGCTCCACCGGATCCTTAaatctggaaggggaagctgc
	PCR III<br>template: splice PCR I+II	TCGAGCGGTGGGAATTCTGGTGGAGGATCCatggtagcacacaatcaag<br><br>AGATCTTCCGCTAGCTCCACCGGATCCTTAaatctggaaggggaagctgc
HiBiT-SOCS1 R188K R193K R201K	HiBiT-SOCS1 WT	TCGAGCGGTGGGAATTCTGGTGGAGGATCCatggtagcacacaatcaag<br><br>AGATCTTCCGCTAGCTCCACCGGATCCTTAaatctggaaggggaagctgctcaggtagtcCTTc<br>agcacggggttcagggggatCTTagccaggttctcCTTgcccacggtg
HiBiT-ODC	HEK 293 cDNA	TCGAGCGGTGGGAATTCTGGTGGAGGATCCatgtctgcggagagcggccc<br><br>CCAGATCTTCCGCTAGCTCCACCGGATCCttaaacgaaaagattttcttc
HiBiT-BRD4	NanoLuc-BRD4	TCGAGCGGTGGGAATTCTGGTGGAGGATCCatgaacaactttggtaatg<br><br>ACCAGATCTTCCGCTAGCTCCACCGGATCCCtacacattaatactagccg

References

[S1] Zengerle, M., Chan, K.-H., and Ciulli, A. (2015). Selective Small Molecule Induced Degradation of the BET Bromodomain Protein BRD4. ACS Chem Biol 10, 1770–1777. 10.1021/acschembio.5b00216.

[S2] Asher, G., Bercovich, Z., Tsvetkov, P., Shaul, Y., and Kahana, C. (2005). 20S proteasomal degradation of ornithine decarboxylase is regulated by NQO1. Mol Cell 17, 645–655. 10.1016/j.molcel.2005.01.020.

[S3] Mathieson, T., Franken, H., Kosinski, J., Kurzawa, N., Zinn, N., Sweetman, G., Poeckel, D., Ratnu, V.S., Schramm, M., Becher, I., et al. (2018). Systematic analysis of protein turnover in primary cells. Nat Commun 9, 689. 10.1038/s41467-018-03106-1.

[S4] Szulc, N.A., Piechota, M., Thapa, P., and Pokrzywa, W. (2023). Data accompanying the manuscript Lysine-deficient proteome can be regulated through non-canonical ubiquitination and ubiquitin-independent proteasomal degradation. 10.5281/zenodo.7545561
