# Supplementary material for: Lysine deserts and cullin-RING ligase receptors: Navigating untrodden paths in proteostasis
Source: iScience. 2023 Oct 28;26(11):108344. doi: 10.1016/j.isci.2023.108344 (PMC10665810; doi:10.1016/j.isci.2023.108344)
Supplement: Data S1. Analysis of lysine deserts’ correlation with protein half-life, related to Figure 7 [file mmc3.zip › Data S1.pdf]

A

Lysine-less region expressed as fraction

Analysis of the whole datasets

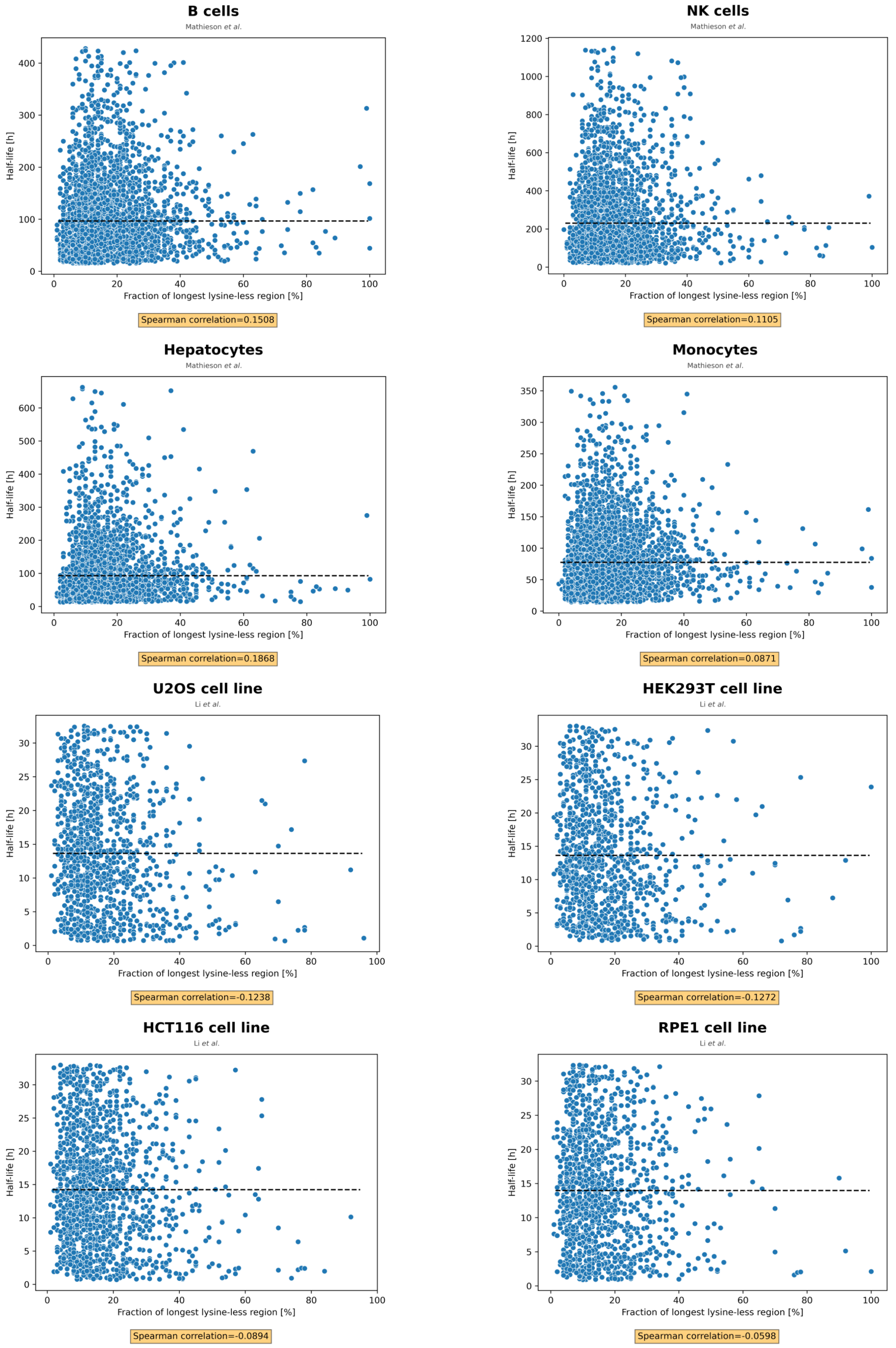

B

Lysine-less region expressed as fraction

Analysis of the 10% of the shortest living proteins from each dataset

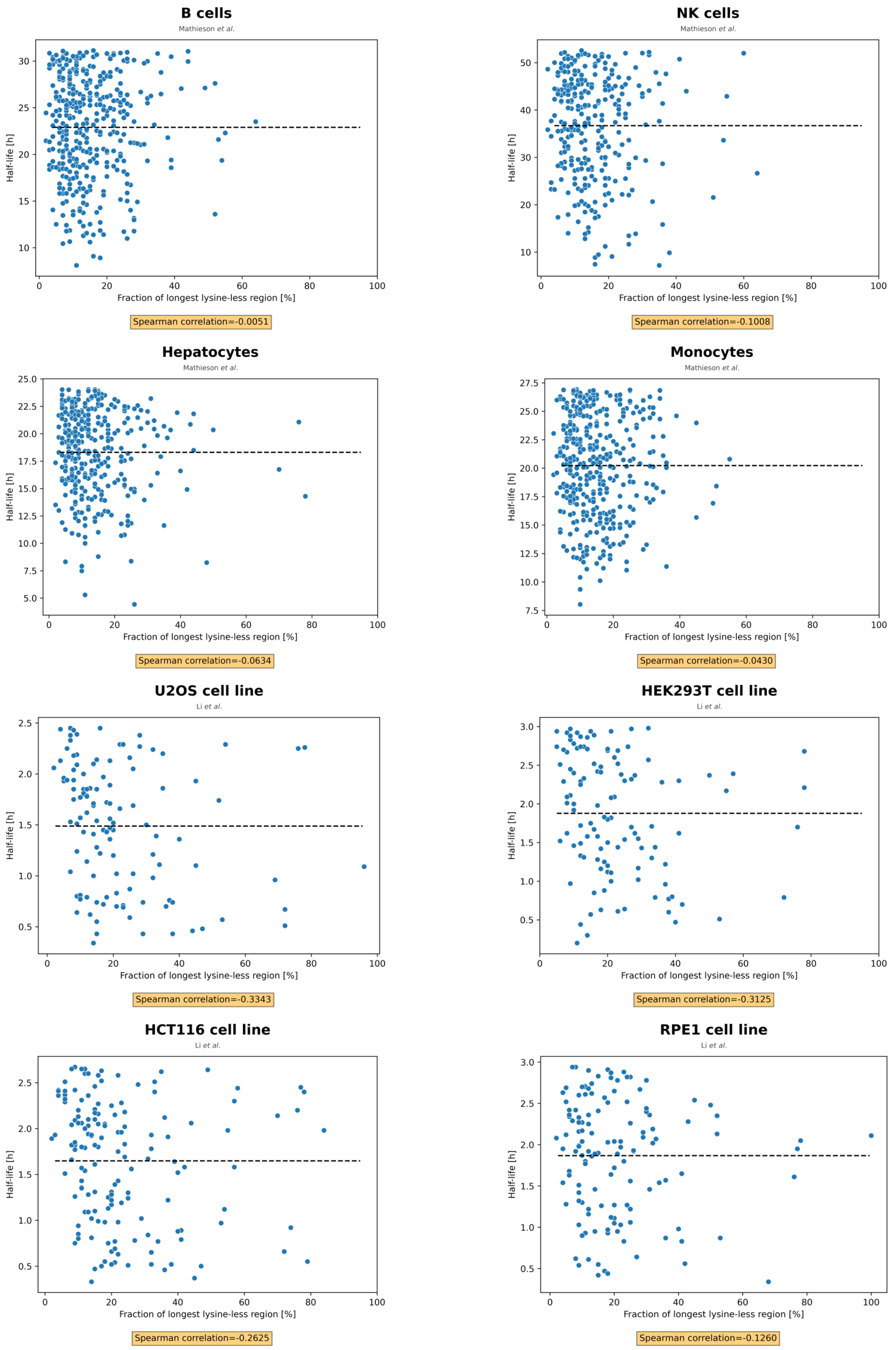

C

Lysine-less region expressed as nominal value

Analysis of the whole datasets

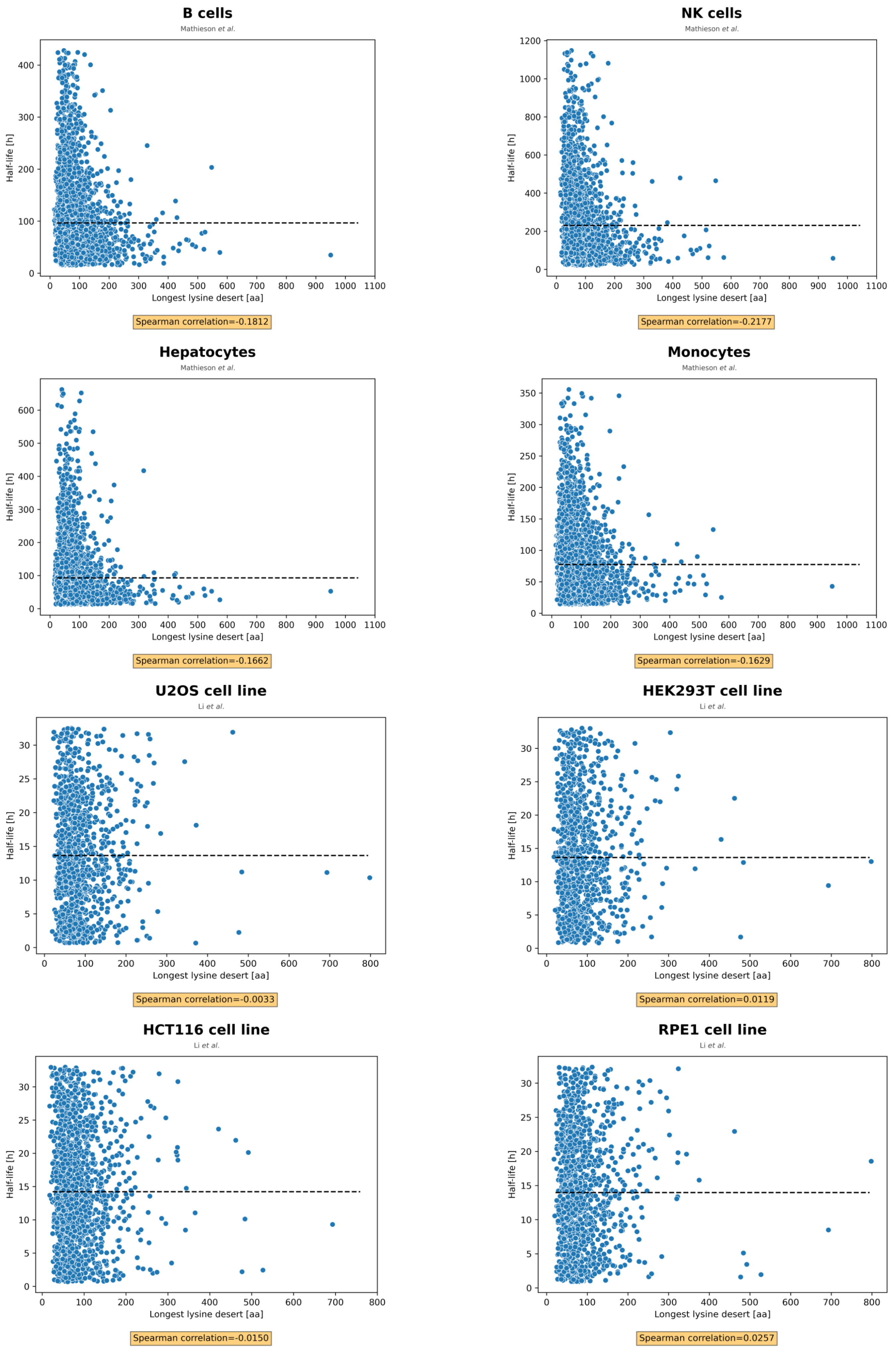

D

Lysine-less region expressed as nominal value

Analysis of the 10% of the shortest living proteins from each dataset

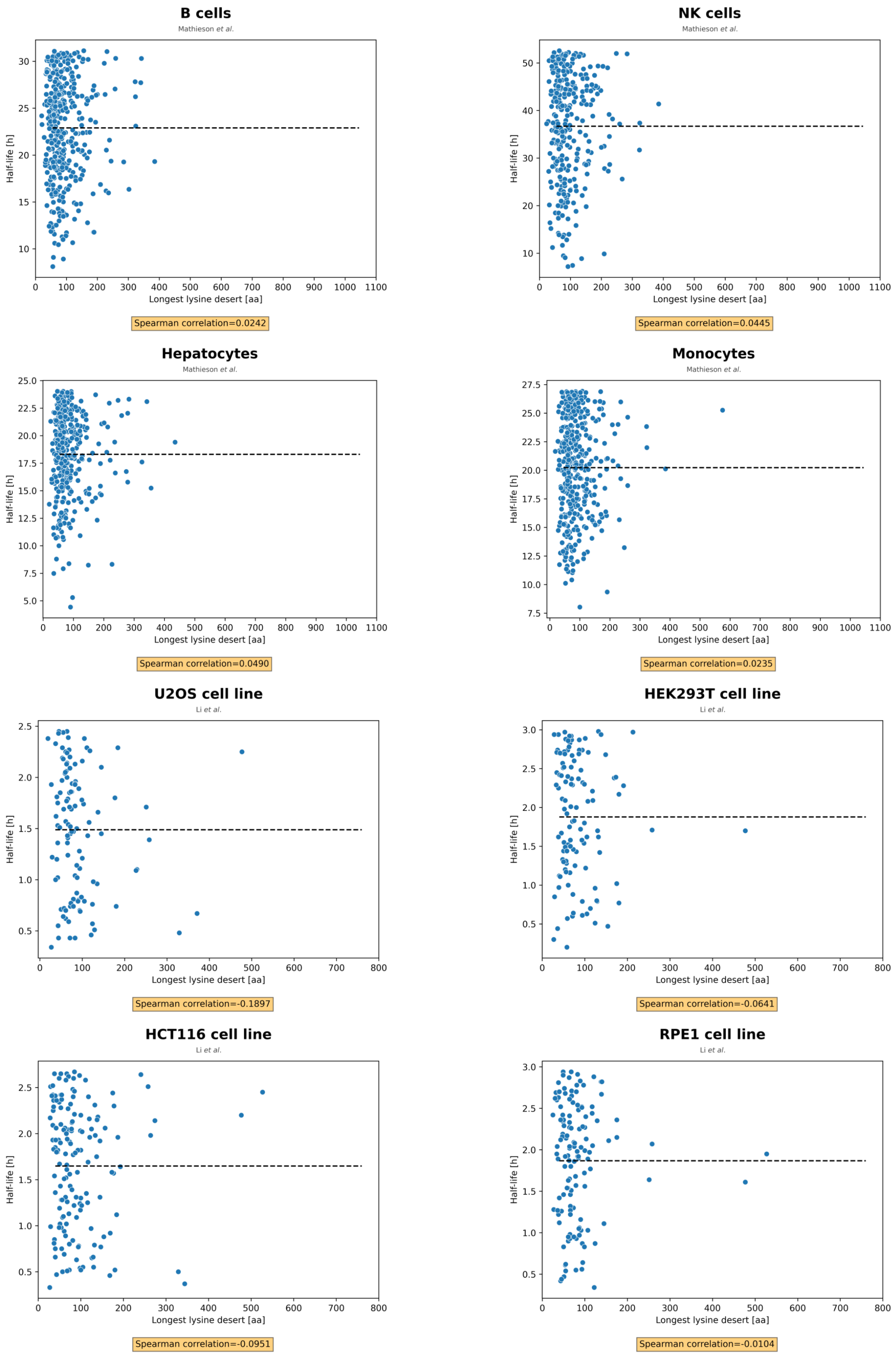

Data S1. Analysis of lysine deserts correlation with protein half-life, related to Figure 7. Scatter plots showing a lack of correlation between the length of the lysine-less region expressed as fraction **(A-B)** or nominal value **(C-D)** and protein half-life in eight different cell types from two large-scale proteomic studies of human proteins [S1-S2]. Panels A and C show analyses for the whole datasets while panels B and D for the 10% of the shortest living proteins from each dataset only. Dashed line indicates mean half-life for each dataset. The Spearman rank-order correlation coefficient is denoted below each plot.

References

[S1] Mathieson, T., Franken, H., Kosinski, J., Kurzawa, N., Zinn, N., Sweetman, G., Poeckel, D., Ratnu, V.S., Schramm, M., Becher, I., et al. (2018). Systematic analysis of protein turnover in primary cells. Nat Commun 9, 689. 10.1038/s41467-018-03106-1.

[S2] Li, J., Cai, Z., Vaites, L.P., Shen, N., Mitchell, D.C., Huttlin, E.L., Paulo, J.A., Harry, B.L., and Gygi, S.P. (2021). Proteome-wide mapping of short-lived proteins in human cells. Mol Cell 81, 4722-4735 e4725. 10.1016/j.molcel.2021.09.015
